# Human Internal Exposures of Bisphenol A and Six Data-Poor Analogs Predicted by Physiologically Based Kinetic Models with Multimodal Parametrization

**DOI:** 10.1021/acs.est.5c00513

**Published:** 2025-09-25

**Authors:** Hélène Bigonne, Amrei Rolof, Inga Potapova, Shana J. Sturla, Georg Aichinger

**Affiliations:** Department of Health Sciences and Technology, Institute of Food Nutrition and Health, 27219 ETH Zürich, CH-8092 Zürich, Switzerland

**Keywords:** bisphenols, BPA, PBK modeling, ADME, pharmacokinetics, endocrine disrupting chemicals

## Abstract

Bisphenols (BP) AF, B, E, F, M, and S are increasingly used as bisphenol A (BPA) substitutes. Despite widespread exposure and potential adverse health outcomes, they are poorly understood in terms of toxicokinetics, i.e., their absorption, distribution, metabolism, and excretion. We thus developed physiologically based kinetic models for different human physiological standards to predict internal concentrations of prevalent bisphenols following oral exposure. To address the imbalances in available human data among these chemicals, we used multimodal parametrization methods, including in vitro measurements of metabolism, computational prediction of gastrointestinal absorption, and rat–human extrapolation of enterohepatic circulation. Then, the models were evaluated against available human toxicokinetic data for BPA and BPS, revealing that 66% of predicted *C*_max_, *t*_max_, and AUC values fell within a 2-fold difference from in vivo measures. Using environmentally relevant exposure levels to compare internal levels of all tested bisphenols, we observed significant differences in the toxicokinetic profiles. Concerning tissues of toxicological concern, BPS had the highest concentration in blood and testes, while BPM accumulated in the thyroid and BPAF in the breasts. The present models are expected to facilitate a more precise evaluation of health risks induced by BPA analogs, guiding their safer use.

## Graphical abstract

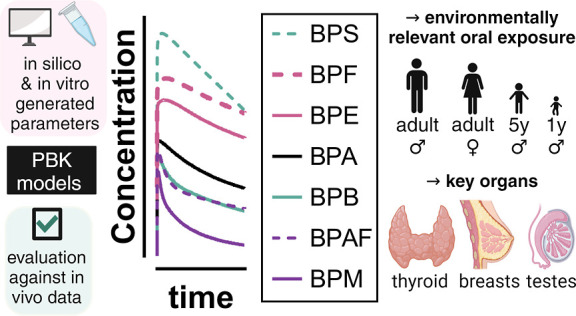

## Introduction

The rigid molecular structure of bisphenol compounds confers essential material properties for plastic production, such as chemical resistance and thermal stability.^1^ In numerous countries,^2–6^ bisphenol A (BPA), once an industry staple, now faces restrictions or bans, leading to an increasing use of its structural analogs as substitutes. BPAF, BPB, BPE, BPF, BPM, and BPS were detected in beverage packaging materials.^7^ Bisphenol substitutes can migrate from packaging materials to their content,^8^ which raises significant concerns, as their structural similarity to BPA suggests they might also exert endocrine disrupting (ED) effects by binding to biological receptors.^6,9^ These interactions can lead to adverse outcomes, notably, reproductive disorders, thyroid and gonadal dysfunction/cancer, breast cancer, obesity, and diabetes.^10^ BPB and BPS have been identified as substances of very high concern under the European REACH Regulation due to their ED properties and reproductive toxicity.^11,12^ BPAF meets the ED criteria for human health^13^ and the opinion of ECHA’s committee for risk assessment on BPAF as toxic to reproduction (Repr. 1B) is in the European Commission for decision-making.^14^

To evaluate the safety of BPAF, BPB, BPE, BPF, BPM, and BPS, Next Generation Risk Assessment (NGRA) principles recommend an exposure-led approach.^15^ This involves converting external exposure data into concentration–time profiles in blood and organs, underscoring the necessity of prior knowledge of absorption, distribution, metabolism, and excretion (ADME) processes to determine relevant points of departure for hazard predictions. Human biomonitoring data confirms the widespread exposure to BPA analogs results in important human exposure, particularly among vulnerable populations, yet fails to provide essential toxicokinetic insights (Table S1).^16–25^

The kinetic behavior of BPA following oral administration has been well characterized.^26,27^ After a rapid and efficient absorption in the gastrointestinal tract, its phase II conjugation in the gut wall and the liver leads to the extensive formation of its main metabolite, BPA glucuronide, which is rapidly eliminated via urine.^26^ To predict chemical concentrations in key organs, further mechanistic insights were provided by physiologically based kinetic (PBK) models. An eight-compartment PBK model capable of predicting human internal BPA exposures was proposed by Yang et al.^28^ It is sometimes assumed that other bisphenols undergo similar ADME patterns,^29^ but since even slight differences in xenobiotic structures can induce significant variations in ADME processes, this application of read-across for their risk assessment should be evaluated. Of note, while the toxicokinetics of BPA^30^ and BPS^31^ have been studied, the fate of BPAF, BPB, BPE, BPF, and BPM was not assessed in humans. This lack of data limits the applicability of the PBK model parametrization method of Yang et al.^28^ to other bisphenols.

Karrer et al.^32^ addressed the challenge of extending the existing BPA PBK model to BPS, BPAF, and BPF. Their approach relied on molecular weight (MW) and structural similarities: BPA and BPF were grouped together, as were BPS and BPAF, to allow the reuse of toxicokinetic measured data from well-characterized bisphenols for calibrating models of data-poor analogs. Although this model extended predictions to less-characterized bisphenol substitutes, its application remains limited to bisphenols with minimal physicochemical differences to BPA and BPS.

To address the lack of human toxicokinetic data for a wider diversity of bisphenols, we constructed PBK models for BPA and six of its structural analogs using the OECD methodology (Figure S1).^33^ We hypothesized that these models could predict internal exposures following oral administration, achieving a level of accuracy comparable to that of previous models developed with calibration to human data.^31,32^ A common concept was established for four standard human models varying by age and sex. The models for each bisphenol were individually parametrized using a multimodal approach, which integrates quantitative structure–activity relationship (QSAR) methods, quantitative in vitro to in vivo extrapolation (QIVIVE), glucuronidation kinetics measured in S9 fractions, and rat PBK modeling. The model performance was evaluated against the human toxicokinetic data. Concentrations in 12 compartments including blood, thyroid, testes, and breasts were predicted after environmentally relevant oral exposure of BPA, BPAF, BPB, BPE, BPF, BPM, and BPS.

## Materials and Methods

### Scope and Purpose of Exposure Scenarios

Diet is the main route of human exposure to bisphenols; therefore, models were designed herein for oral administration.^34^ Several exposure scenarios were defined for parameter gathering, model evaluation, and internal exposure assessment (Table 1). In scenarios 1 and 2, a rat PBK model was used to parametrize enterohepatic circulation (EHC) rates for application in human PBK models. Scenarios 3 and 4 replicated the administration of BPA from Teeguarden et al.^30^ and BPS from Oh et al.^31^ for model evaluation. To illustrate potential BPA replacement by BPAF, BPB, BPE, BPF, BPM, and BPS, scenarios 5 to 12 assumed consistent exposure rates among all these bisphenols, based on the 2015 EFSA assessment of BPA external exposure.^35^ Models were developed for four human physiologies: male (“man”) and female adults (“woman”), male “child”, and male “toddler” (Table S2). These models were selected based on available physiological data for key organs (thyroid, breasts, and testes), related to ED and the detected bisphenol exposure level. The repeated dose scheme from Karrer et al.^32^ was reproduced (scenarios 9 to 12) to model chronic exposure via three meals a day.

**Table 1:** Exposure Scenarios, Scope, and Purpose of Modeling

scenario	purpose	model	BP	dosing	key organ(s)
1	EHC determination	rat	BPAF	single dose^a^: 340 mg/kg bw	blood
2	EHC determination	rat	BPF	single dose^a^: 200 mg/kg bw	blood
3	model evaluation	man^b^	BPA	single dose^a^: 30 μg/kg bw	blood
4	model evaluation	adult^c^	BPS	single dose^a^: 8.1 μg/kg bw	blood
5	internal exposure assessment	man	^d^	single dose^a^: 336 ng/kg bw	blood, thyroid, and testes
6	internal exposure assessment	woman	^d^	single dose^a^: 389 ng/kg bw	blood, thyroid, and breasts
7	internal exposure assessment	child	^d^	single dose^a^: 818 ng/kg bw	blood, thyroid, and testes
8	internal exposure assessment	toddler	^d^	single dose^a^: 869 ng/kg bw	blood, thyroid, and testes
9	internal exposure assessment	man	^d^	repeated dose^e^: 112 ng/kg bw	blood, thyroid, and testes
10	internal exposure assessment	woman	^d^	repeated dose^e^: 130 ng/kg bw	blood, thyroid, and breasts
11	internal exposure assessment	child	^d^	repeated dose^e^: 273 ng/kg bw	blood, thyroid, and testes
12	internal exposure assessment	toddler	^d^	repeated dose^e^: 290 ng/kg bw	blood, thyroid, and testes

a Single oral dosing at *t* = 0 h. Total duration of the simulation: 48 h.

b BW and H adjusted to the population (10 men) of the toxicokinetic study by Teeguarden et al.^30^

c BW and H adjusted to the population (4 men and 3 women) of the toxicokinetic study by Oh et al.^31^

d BPA, BPAF, BPB, BPE, BPF, BPM, and BPS.

e Repeated oral dosing at *t* = 0, 6, 12, 24, 30, 36, 48, 56, 60, 72, 78, and 84 h. Total duration of the simulation: 96 h.

A common model structure was employed for the seven bisphenols (Figure 1). When not individually compartmentalized, organs were categorized as either slowly perfused tissues or rapidly perfused tissues and combined. Compartments related to the thyroid, testes, and breasts (depending on sex) were included in human but not rat models. Code was compiled in Berkeley Madonna 10 and Python (see Supporting Information and GitLab: https://gitlab.ethz.ch/eth_toxlab/pbk_model_bisphenol_analogs).

**Figure 1:**
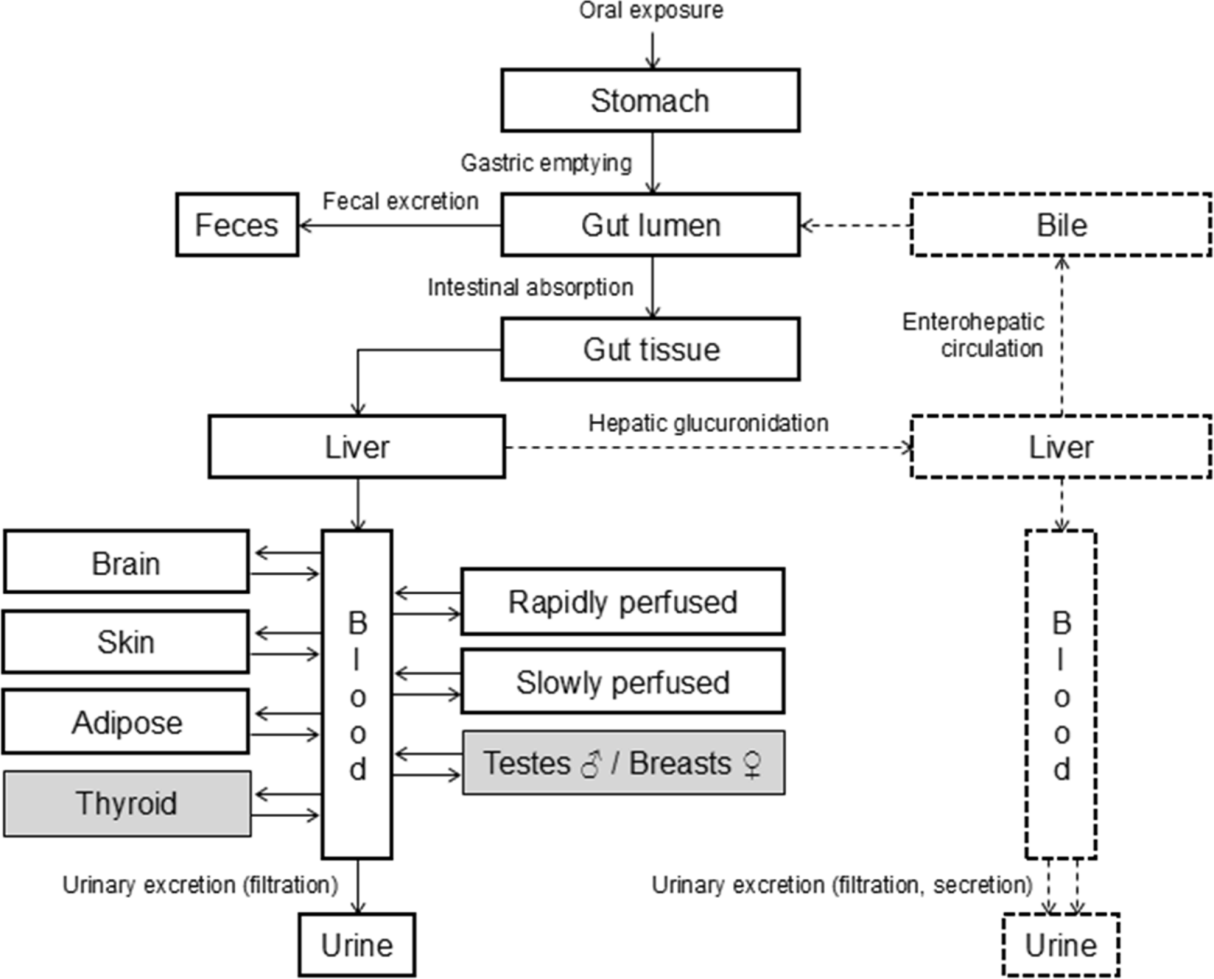
Schematic PBK model concept for BPA analogs (flows and compartments with continuous lines) and their glucuronidated metabolites (flows and compartments with dashed lines). Compartments with gray filling are only present in human models.

### Multimodal Parametrization of PBK Models

These PBK models were parametrized using a multimodal framework that integrates new approach methodology tools into a reproducible chemical-family wide workflow. This framework combines QSARs, in vitro experiments, and cross-species extrapolation to parametrize PBK models for a family of compounds, some of which were considered data-poor (Figure 2). These methods were chosen for their simplicity of use, with QSARs, for example, requiring minimal prior information such as molecular structures, and for their compatibility with an iterative verification process in which predictions were compared to previously available results to ensure the biological accuracy of the parameters. By harmonizing the parametrization process in an in vivo-data-independent manner across an entire compound class, this multimodal framework ensures methodological consistency, reproducibility, and applicability to other families of compounds. Parameters related to physicochemical characteristics of seven bisphenols and their glucuronides were set to the mean of values predicted from their chemical structures with ChemDraw 20.0 and Chemaxon Playground v1.6.2 (Figure 2 and Tables S3 and S4). Organism-based parameters were tailored to account for species, sex, and age when possible (Figure 2 and Table S2).^36–49^ Parameters based on both the organism and chemical were partition coefficients, intestinal uptake, EHC rate, and glucuronidation kinetics (Figure 2).

**Figure 2:**
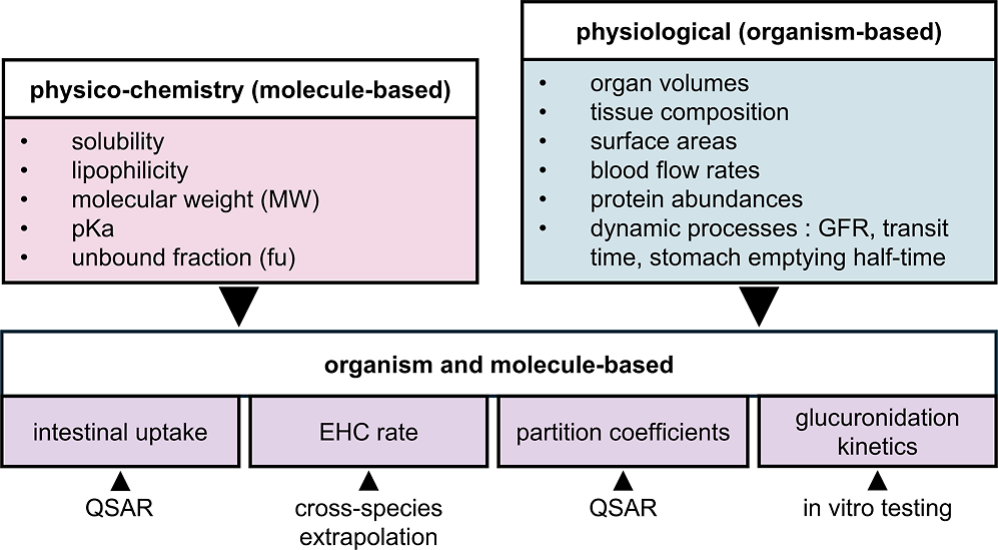
Overview of parameters used in the present model and how they were acquired.

When possible, partition coefficients and plasma unbound fractions were calculated with the QIVIVE toolbox,^50^ using the methods of Rodgers and Rowland^51^ and of Lobell and Sivarajah,^52^ and used for different ages, sex, and species (Supporting Information Table S5). For combined compartments, weighted sums of partition coefficients for the constituent organs were used. Partition coefficients of thyroid and testes were calculated using data from Pilari et al.^53^ Based on Ulaszewska et al.,^54^ breasts partition coefficients were calculated as the product of adipose partition coefficients and fat volume in breasts (24.5%).^55^

To parametrize the intestinal uptake (*k*_a_) of all bisphenols, Caco-2 apparent permeability coefficients (*P*_app_) were only available for BPA, BPF, and BPS,^56^ so two QSARs were additionally used to predict *P*_app_ of the seven bisphenols, here referred to as “QSAR K” (Kamiya et al.^56^) and “QSAR L” (Lanevskij and Didziapetris).^57^ LogD (lipophilicity) values were computed using the Chemaxon predictor,^58^ considering pH values of 6.0 (apical) and 7.4 (basal). For “QSAR L”, standard values were set for pH (7.4), stirring rate (0 rpm), and average pore radius (5 Å), and McGowan characteristic volumes were calculated according to Zhao et al.^59^ Then, all available *P*_app_ values per bisphenol were averaged. The scaling algorithm of Sun et al.^60^ was applied (“all drugs” regression; pH 7) to obtain the in vivo effective permeability (*P*_eff_) per intestinal surface area.

Glucuronidated bisphenols were assumed to undergo EHC and renal secretion, both relying on active transporters present in the liver and the kidney. We modeled these processes using a common parameter, “EHCr” (EHC rate), to describe them. We modeled EHC by simulating hepatic glucuronidated bisphenol cycling through bile into the gut lumen, where it reverts to its parent form (Figure 1). In this context, EHCr represents the portion of metabolite leaving the liver to undergo biliary excretion. Then, bile transfer from the bile compartment to the gut lumen is assumed to be immediate and complete (Figure 1). Renal secretion was represented from the blood compartment, factorized by the blood flow rate to the kidneys, EHCr representing here the transport via efflux pumps, scaled to the renal context by the ratio of volume between the kidneys and the liver, assuming comparable expression levels.^61^ The value of the EHC rate was constrained between 0 and 1 and fitted as described.^62^ Predicted-to-observed *C*_max_ ratios in blood were calculated by adjusting EHCr until rat blood concentrations matched values from animal studies for BPF^63^ or BPA^48^ at 24 and 48 h, respectively, while maintaining the *C*_max_ ratio. EHCr values for other bisphenols were calibrated by MW,^64^ with BPAF as the lightest and BPF the second heaviest. For simplicity, the EHCr of BPM was set equal to BPAF. Human models of the same sex used the same values, assuming stable biliary export protein expression.^65^

### Measurement and Parametrization of Glucuronidation Kinetics

The glucuronidation kinetics of BPA, BPB, BPE, BPF, BPAF, and BPM were determined as described in the Supporting Information (Tables S6 and S7). Outliers were excluded using the Nalimov test.^66^ Glucuronidation rates were calculated from compound loss over time using GraphPad Prism 10.2.3 (2024), with Michaelis–Menten and substrate inhibition fitting algorithms applied, selecting the best fit based on maximal R^2^.

Rates were scaled by in vitro context: human hepatic S9 fractions with 107.3 mg/g liver,^67^ rat hepatic S9 with 143 mg/g liver,^68^ and human hepatic microsomes with 32 mg/g liver.^32,69^ To account for age-related UDP glucuronosyltransferase (UGT) 2B15 protein expression, ontogeny scaling established a scaling factor for glucuronidation (SF_g_) kinetics (eq 1).^70^ Protein abundances were based on Bhatt et al.,^71^ with both child and toddler models classified under early childhood.

1 $${SF}_{g}=\frac{protein\;abundance\;in\;age\;category\;of\;interest\;\left(\frac{pmol}{\mathrm{microsomal protein}}\right)}{protein\;abundance\;in\;adult\;\left(\frac{pmol}{\mathrm{microsomal protein}}\right)}$$

### Model Evaluation

The performance of rat and human PBK models was evaluated by comparing predicted blood *C*_max_, *t*_max_, and AUC values to their in vivo measured values, for BPAF,^48^ BPF,^63^ BPA,^30^ and BPS.^31^ Toxicokinetic parameters predicted for BPA, BPA glucuronide, BPS, and BPS glucuronide were also compared to previous modeling results.^31,32^

Additionally, to evaluate the importance of excretion routes in the model, mass balance analysis was implemented within the model code. This analysis quantified the fractions of the dose excreted via urine and feces, at 12, 24, and 48 h following a single-dose exposure. The resulting excretion fractions were then compared with reported experimental data.^30,72^ To evaluate the balance between renal and biliary elimination pathways in the PBK models, clearance values were derived from the outputs of the male model exposed to BPA (exposure scenario 5, Table 1). Total renal clearance (CL_R_) was defined as the sum of parent BPA renal clearance (resulting from filtration) and BPA glucuronide renal clearance (including filtration and secretion). These clearances were calculated as the ratio between the blood AUC of either BPA or BPA glucuronide over 24 h and the corresponding cumulative urinary excretion within the same time frame. Biliary clearance (CL_bile_) was estimated as hepatic blood flow (QL) multiplied by EHCr. According to Derendorf and Schmidt,^73^ the bile-to-plasma concentration ratio was derived as CL_bile_ divided by daily bile flow, set to 700 mL/day.^40^ To further assess the impact of EHC on systemic exposure, the apparent fraction absorbed by BPA was estimated as the ratio of the product of total clearance (renal plus biliary) and blood AUC to the oral dose, accounting for renal and biliary elimination pathways.

### Sensitivity Analysis

The impact of parameter deviation on model predictions was assessed by local and global sensitivity analysis (GSA).The local sensitivity analysis (LSA) was performed in human models for all bisphenols, based on the method by Evans and Andersen^74^ as described by Aichinger et al.^62^ A parameter with an absolute sensitivity coefficient > 0.1 was considered sensitive. The GSA was performed in all human models, according to the workflow of McNally et al.,^75^ consisting in the Morris screening exercise on all parameters, followed by the extended Fourier amplitude sensitivity test (eFAST) on a selected subset of parameters. To investigate parameter sensitivity in the distribution and elimination phase, the GSA was conducted on AUC between the times 0 and 4 h of simulation, using the package SAlib.^76^ Parameter ranges were set to cover approximately ±2 standard deviations from the initial value, based on the coefficient of variation estimated from the literature (Table S8).^44,49,71,77–81^

### Uncertainty Analysis

To address parameter uncertainty in concentrations that were predicted with human PBK models, 10,000 Monte Carlo (MC) simulations were performed. Each sensitive parameter was resampled according to probability distributions from the literature (Table S8).^44,49,71,77–81^ To ensure physiological plausibility, EHC rates were constrained between 0 and 1, and the sum of all relative organ weights and the sum of all relative organ blood flows were kept constant. MC simulation outcomes were analyzed by comparing the first quartile, the median, and the third quartile values of concentrations per time point.

## Results and Discussion

### PBK Model Parametrization without In Vivo Data

The parametrization of intestinal uptake could be achieved by combining various techniques. We compared *P*_app_ values of the BPA analogs, either measured in Caco-2 cells monolayers (BPA, BPF, and BPS) and/or predicted by two QSARs (all analogs) in Figure S2.^56,57^ Predictions were consistent between the applied QSARs for BPA, BPAF, BPB, BPE, and BPF, ranking the permeability in the same order, with values differing by a maximum of 11 × 10^–6^ cm/s. Moreover, computed and in vitro measured transport values were in line for BPA and BPF. This was not observed, however, for BPM and BPS. For BPM, QSAR L predicted lower permeability than that predicted with QSAR K. This could be attributed to differing sensitivities of the QSARs to the comparably larger molecular volume, which is considered in QSAR L. For BPS, the two QSAR-predicted *P*_app_ values aligned closely but were lower than reported in vitro permeability. Furthermore, the inclusion of three bisphenol compounds in the training data set of QSAR K^56^ did not significantly enhance the predictions of *P*_app_ values compared to QSAR L,^57^ indicating specialized training sets may not necessarily improve accuracy. These findings highlight the feasibility of using QSARs for modeling intestinal uptake without use of error-prone read-across while also demonstrating the value of integrating multiple methods and in vitro measurements to reveal potential biases in in silico approaches.

To develop a common model for all bisphenols using molecule-specific parameters, the parametrization of hepatic metabolism was based on glucuronidation kinetics measured in subcellular liver fractions. Glucuronidation kinetics were de novo measured in human liver S9 fractions for BPA, BPB, BPE, and BPM, while for BPS, BPF, and BPAF, previous kinetic data from hepatic microsomes^32^ were used (Supporting Information Figures S3 and S4 and Supporting Information Table S9). Data generated from those various experiments was considered homogeneous since compositions of homologous UGTs are strongly correlated between commercially available liver microsomes and S9 fractions.^82^ Among BPA analogs, BPB was glucuronidated the most and BPS the least efficiently (Figure S3). Of note, BPA glucuronidation in human hepatic S9 fractions in the present study was faster than that previously reported in microsomes. To the best of our knowledge, glucuronidation kinetics for BPB, BPE, and BPM has not been previously reported. Intestinal glucuronidation of BPA, BPS, BPAF, and BPF was previously characterized in human intestinal microsomes and cell lines,^32,83–85^ even though bisphenols are metabolized predominantly in the liver.^32^ In line, we observed negligible glucuronidation rates in small intestinal S9 fractions only for BPA, BPB, BPE, and BPM. Intestinal glucuronidation could not be characterized for all bisphenol analogs and was not included in the model. Hepatic sulfation was also not represented due to the lack of comprehensive data for all analogs.^86^ However, the latter might have a significant importance in children, particularly in girls,^70^ which is unfortunately beyond the scope of the present model.

Rates of transport via efflux pumps were introduced as the parameter “EHCr” and fitted by incrementally adjusting this parameter in rat models until the predicted blood concentrations matched in vivo measurements at later time points. The resulting values were then directly used in human models (Table S10). Thereby, a similar high EHCr rate was predicted for each bisphenol, with more than 63% of glucuronidated bisphenols leaving the liver through biliary excretion. Regarding sex differences, EHCr values were slightly higher in females due to higher BPAF concentrations measured in female rats 24 h post administration.^48^ Additionally, the half-life of BPF was longer in female rats compared to that in males.^63^

Tissue-to-blood partition coefficients were estimated using a uniform approach based on an established QSAR method (Table S5).^50,51^ As discussed for a previous PBK model for some bisphenols,^32^ lipophilicity had an important influence on partition coefficient values. Overall, in a data-poor context, determining partition coefficients is largely hindered by the lack of tissue composition data available in the literature. Further characterization of sensitive endocrine tissues, such as the ovaries, breasts, and uterus, is needed to enhance the predictive scope of models used in female reproductive toxicity risk assessment.^87,88^

The present PBK model incorporates 52 parameters, among which 14 depend directly or indirectly on logP and p*K*_a_ (namely, partition coefficients, Papp, and EHCr). These physicochemical properties were predicted from molecular structures using two independent software tools (ChemDraw 20.0 and Chemaxon Playground v1.6.2.). Given the uncertainty inherent in such in silico predictions, we enhanced model robustness by averaging the values obtained from both methods (Tables S3 and S4). While this approach reduces potential biases, differences in physicochemical inputs can still influence PBK model outcomes, highlighting the importance of careful selection and validation of these parameters.

Finally, the multimodal parametrization strategy, while designed for broad applicability across structurally related compounds, also facilitates future refinement. As new experimental or computational data become available, individual parameters can be re-evaluated without altering the model’s overall structure. For example, dermal exposure to bisphenols (which may be of equal or even higher toxicological relevance than dietary exposure)^89–91^ could not be included at this stage due to the lack of transferable and compound-specific input data, but may be considered in future model expansions.

### PBK Model Evaluation

The performance of the present model in predicting blood concentrations of BPA, BPS, and their metabolites was evaluated by using human experimental data. Moreover, the distribution of predicted concentrations obtained by resampling sensitive parameters in MC simulations, to account for parameter variability and uncertainty, was compared with the observed ranges of measured values for blood *C*_max_ (BPA, BPA glucuronide, BPS, and BPS glucuronide), *t*_max_ (BPA and BPA glucuronide), and AUC (BPA, BPA glucuronide, and BPS glucuronide).

Concerning BPA, the present model was evaluated with measures from Teeguarden et al.,^30^ and predicted blood *C*_max_ values for BPA and its glucuronide were on average 63% and 80% of the observed values, respectively (Table 2). In the present predictions, *t*_max_ for BPA was lower than the observed value, while *t*_max_ for BPA glucuronide was similar. Predicted AUC for BPA and predicted AUC for BPA glucuronide were both about 1.5 times higher than those observed. Five of six predicted values were within the measurement ranges, and the range of predictions predicted by MC simulations overlapped with all measured ranges. These results indicate that the present model generates reliable predictions of the BPA concentrations.

**Table 2:** Comparison of Measures and Predictions of Toxicokinetic Parameters for Oral Ingestion of BPA or BPS

	BPA	BPA glucuronide	BPS	BPS glucuronide
*C*_max_ (nM)				
in vivo	0.43 (0.3–0.7)^a^	286 (173–386)^a^	8.79 (6.07–11.79)^d^	10.53 (7.77–14.2)^d^
present model^b^	0.27 (0.15–0.47)	228 (168–297)	14.1 (9.04–21.20)	29.2 (18.6–42.8)
previous model	1.31 (0.89–1.66)^c^	610 (580–648)^c^	10.1 (7.96–12.24)^e^	
*t*_max_ (h)				
in vivo	1.6 (0.5–2.2)^a^	1.2 (0.8–2.2)^a^	0.5 (0.25–0.5)^d^	1.0 (1.0–1.0)^d^
present model^b^	0.70 (0.61–0.93)	1.16 (1.00–1.31)	2.15 (1.9–2.3)	1.0 (0.99–1.25)
previous model	0.72 (0.67–0.77)^c^	0.94 (0.93–0.97)^c^	0.57 (0.26–0.88)^e^	
AUC (nM x day)				
in vivo	2.5 (1.4–5.7)^a^	680 (571–1′210)^a^	40 ^31–49^ ,^d^	78.7 (54–103)^d^
present model^b^	3.97 (2.38–6.64)	1′036 (710–1′511)	163 (100–264)	191.3 (125–284)
previous model	4.15 (2.91–5.15)^c^	1′111 (1′050–1′210)^c^	39.8 ^37–43^ ^,^ ^e^	

a Concentrations measured in 10 men by Teeguarden et al.,^30^ following administration of 30 μg/kg bw d6-BPA in 355 mL of soup.

b Intervals of variability of the present model predictions were set as the first and third quartiles of the MC simulations.

c Previously modeled by Karrer et al.^32^

d Concentrations measured after administration of 8.75 μg/kg bw of d4-BPS in a chocolate cookie to 4 men and 3 women.^31^

e Previously modeled by Oh et al.^31^

The present model was also evaluated for BPS, using measures from Oh et al.^31^ Predicted blood *C*_max_ values for parent and glucuronide were on average 1.6 and 2.8 times higher than in vivo observed values, respectively. The average predicted *t*_max_ was delayed by 1.7 h for the parent compound and very similar to the measure for the metabolite. The mean predicted AUC value for BPS glucuronide was about 2.4 times higher than the in vivo value, while the mean predicted AUC for BPS parent was 4 times higher than observed.

Furthermore, the performance of the present model was compared to previous modeling efforts for BPA and BPS to assess the reliability of predictions using different parametrization approaches. The present model more accurately predicted internal bisphenol concentrations than the model developed by Karrer et al.^32^ for five out of six analyzed toxicokinetic parameters (average predicted values for *C*_max_, *t*_max_, and AUC for BPA and BPAg), suggesting superior performances of the present model for BPA (Table 2).^31,32^ Although predictions for BPS toxicokinetics were less accurate than those from the model by Oh et al.,^31^ the latter was built by fitting parameters directly to human experimental data. While this ensures close reproduction of observed concentrations, it may lead to overfitting and limits the predictive value beyond the specific test conditions. In contrast, the present model was constructed from independent biological and chemical data, which offers predictive capabilities beyond the calibration context and supports extrapolation to untested scenarios. For both BPA and BPS, comparisons of predicted blood *C*_max_ values demonstrated that the present multimodal parametrization approach achieves equal or greater similarity to in vivo measures compared to in vivo data fitting methods used in previous models (Table 2). Moreover, since models built in data-poor situations cannot be calibrated, their predictions are limited by increased uncertainty. For instance, the PBK model established by Karrer et al.^32^ was calibrated with in vivo blood concentration values.^31^ An important aspect consists of testing the calibrated model against data that were not used to calibrate the model. Therefore, the present PBK model is the first to be evaluated for two different bisphenols and their metabolites, which ensures more reliable predictions.

The relative importance of excretion routes was evaluated through mass balance analysis (Table S11). Model predictions align closely with experimental data from Thayer et al.^72^ and Teeguarden et al.^30^ Thayer et al.^72^ reported that 94% (range: 86–98%) of total BPA was excreted within 12 h post exposure, while our model predicts 97% and 96% urinary excretion in men and women, respectively. At 24 h, urinary excretion reached 98% (range: 92–100%) in Thayer et al.^72^ and 104% (range: 90–116%) in Teeguarden et al.;^30^ our model estimates 99% for adult models of both sexes. By 48 h, Thayer et al.^72^ observed 80–110% cumulative urinary excretion, while the present model predicts 99%. Regarding unconjugated BPA, Teeguarden et al.^30^ and Thayer et al.^72^ reported urinary fractions of 0.03% and 0.029%, respectively; the present model predicts around 0.01%. These results support the model’s accuracy in reproducing urinary kinetics and excretion profiles.

Calculated renal and biliary clearance values indicate that CL_R_ was 10.1 L/h, while CL_bile_ was 17.0 L/h, yielding a CL_bile_/CL_R_ ratio of 1.7. The corresponding bile-to-plasma concentration ratio was ∼586. Such a prominent biliary route is physiologically plausible, as biliary clearances of 30 L/h or higher can be achieved, and bile-to-plasma ratios can approach 1000 for compounds that are strong substrates of apical efflux transporters.^73^ High biliary clearance is particularly expected for glucuronides, which are polar and ionized (p*K*_a_ ∼ 3) and have molecular weights of >350 g/mol,^73^ properties shared by all metabolites considered in the present work. Additionally, the apparent fraction absorbed for BPA was 2.61, exceeding 1, which is consistent with extensive EHC. Nevertheless, it should be noted that EHC was scaled from rat in vivo data, which may not fully reflect human physiology. As such, while the predicted clearance values are physiologically plausible, it remains to be confirmed whether such high biliary clearance occurs in reality and whether this is specific to bisphenols.

Model evaluation also highlighted the need to improve the parametrization of ADME processes downstream of the systemic distribution. Indeed, all AUC values were overestimated compared to in vivo measures (Table 2). Overestimation of EHC is a primary hypothesis to explain the reduced predictive performance of the present model, especially for BPS, where we observe both an overestimation of the AUC and a delayed *t*_max_. The EHC rate significantly impacted all of the concentrations analyzed with LSA (Figure S5), which aligns with its well-established influence on xenobiotic retention within organisms. However, it was not identified as an influential or interacting parameter in GSA (Figures S6 and S7). The motivation to model an EHC rate from rat toxicokinetic data measured in rats stemmed from the availability of respective blood concentration measurements beyond 24 h post administration and the confirmed existence of EHC for bisphenols in these animals.^27^ However, interspecies differences in EHC have previously been observed between rodents and humans and may explain the suspected inaccuracies in the EHC modeling in the present model. Particularly, MWs of three out of seven bisphenol glucuronides are between the species’ thresholds for excreting chemicals via bile (Table S4), which is estimated to be 400 Da for rats and 475 Da for humans.^64^ Moreover, while the prolonged elimination time of BPS suggests it undergoes EHC in humans,^32^ this observation was not described for other bisphenols that might not even be substrates for human hepatic canalicular transporters.^92^ Ultimately, the EHC rates used in the present model were higher than those of previous estimations. In Yang et al.,^28^ only ten percent of glucuronidated BPA underwent EHC, a rate seven times lower than our predictions (Table S10). Despite the perfectibility of EHC parametrization, we still considered the present approach to be acceptable as it resulted in AUC predictions that were closer to in vivo measurements than those obtained using lower EHC estimates (Table 2).^32^

In the absence of tissue-specific toxicokinetic data in humans (which cannot ethically or practically be obtained under controlled exposure conditions), tissue concentration predictions generated by the present model were compared to available human biomonitoring data (Table S12).^21,22,93–99^ These comparisons aim to assess the general consistency of model predictions with reported concentrations of bisphenol analogs in various biological matrices and tissues while acknowledging the significant limitations inherent to such comparisons. Overall, model-predicted tissue concentrations were lower than human biomonitoring levels by approximately 2 orders of magnitude across blood and solid tissues, whereas urinary concentrations were overpredicted relative to population measurements. Together with the exploratory nature of our comparison, these discrepancies highlight gaps between our simulations and real-world exposures as well as the influence of interindividual variability. They also suggest that alternative parametrizations of EHC may be needed to better reproduce observed tissue concentrations.

A potential limitation of the PBK models is their incomplete representation of the human population. Despite efforts to broaden the applicability of the present models by considering physiological differences specifically for adult men and women and male children and toddlers, some population groups are not considered, e.g., elderly individuals, pregnant women, and those with liver or kidney impairments. While age-related scaling of UGT2B17 and MRP2 expression and MC simulations introduce variability, genetic polymorphisms and other sources of interindividual differences are not fully captured. Exposure was simulated via three daily meals using average EFSA doses over 96 h, which does not reflect lifelong, intergenerational, diverse environmental exposures, or potential mixture effects with other endocrine disruptors.^100^ Geographic, socioeconomic, and occupational factors, as well as vulnerable populations, were also not considered, meaning predictions represent average conditions rather than the full human population.

### Sensitivity Analysis of Model Parameters

In LSA, seven parameters significantly influenced bisphenol concentrations across all key organs and human physiological models: body weight, cardiac output, ECHr, and hepatic glucuronidation kinetics (*V*_max_, *K*_m_, liver volume, and liver blood flow; Figure S5). The scaling factor to account for age differences in glucuronidation kinetics (SFg) was also found sensitive in all non-adults analysis, supporting the use of in vitro over in silico metabolism parametrization for greater reliability. Frequently sensitive parameters also included the gastric emptying half time (GEst) and the blood debit to slowly perfused organs (QSL).

GSA confirmed that model uncertainty is primarily driven by the distribution of hepatic glucuronidation kinetics values. The Morris screening test yielded results consistent with the LSA, identifying *V*_max_, GEst, liver blood flow, and liver volume as influential parameters across all models, with SFg also being important in child and toddler models (Figure S6). These findings are consistent with the expected sensitivity due to the non-linearity of Michaelis–Menten kinetics. Further quantification of main and interaction effects using eFAST revealed that variance in model outputs is largely driven by interactions between parameters (Figure S7).

### Internal Exposure Assessments

Concentrations of BPA, BPAF, BPB, BPE, BPF, BPM, and BPS were predicted in the blood, thyroid, testes, and breast tissues of man, woman, toddler, and child models following single or repeated environmentally relevant oral exposure. For all, higher concentrations were predicted in blood, thyroid, and testes in non-adult models compared to adult models (Figures 3 and S8 and Table 3), which aligns with previous predictions.^32^ Of note, internal maximal concentrations in children and toddlers are particularly increased due to the application of an age-related scaling factor (SFg) for UGT2B15 enzyme expression and correspondingly reduced glucuronidation.^70,71^ For repeated exposure, we predicted bisphenol accumulation in compartments and the attainment of the steady state over the course of 96 h, except for BPM and BPAF in the thyroid, testes, and breasts (Figures 3, 4, and S8 and Table 3).

**Figure 3:**
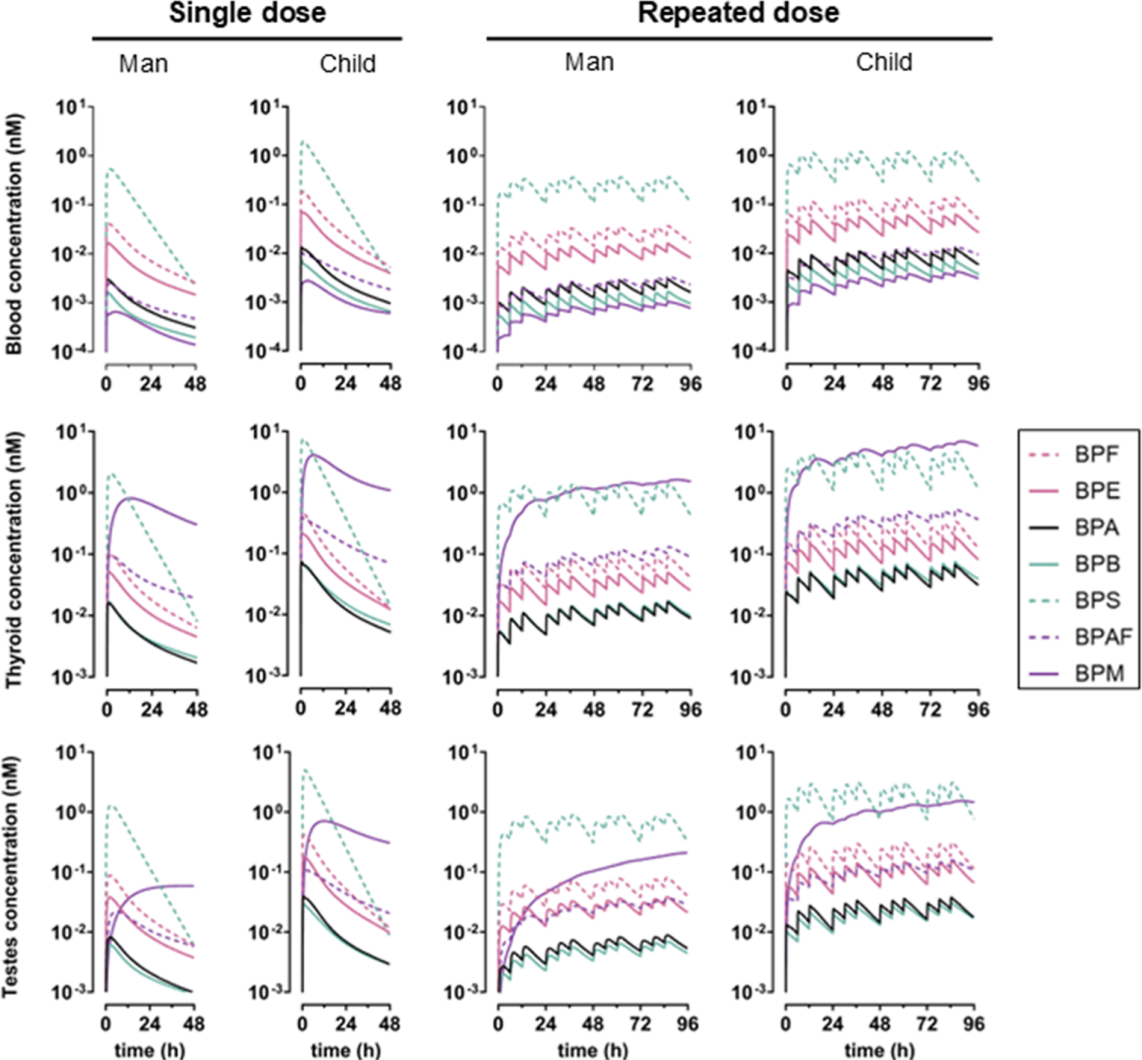
Predicted concentrations profiles (median of 10’000 MC simulations) of BPA, BPAF, BPB, BPE, BPF, BPM, and BPS in blood, thyroid, and testes in man and child models after single or repeated dose (exposure scenarios 5, 7, 9, and 11 of Table 1). Bisphenols are ordered by ascending MW. Since results are very similar between age groups, only man and child model results were represented for simplicity.

**Table 3:** Highest *C*_max_ Values per Bisphenol and per Key Organ after Repeated Dose (Exposure Scenarios 9 to 12), among the Human Models^a^

bisphenol	max. *C*_max_ blood (pM)	max. *C*_max_ breasts (pM)	max. *C*_max_ testes (pM)	max. *C*_max_ thyroid (pM)
BPS	1221^b^ ^,^ ^c^	149^d^	3120^b^ ^,^ ^e^	4658^c^
BPF	140^c^	163^d^	313^c^	351^c^
BPE	62^e^	133^d^	154^e^	192^e^
BPAF	14^g^	244^d^ ^,^ ^f^	157^e^ ^,^ ^f^	547^e^ ^,^ ^f^
BPA	13^g^	45^d^	38^e^	68^e^
BPB	7^g^	45^d^	32^e^	76^e^
BPM	4^g^	335^b^ ^,^ ^d^ ^,^ ^f^	1536^e^ ^,^ ^f^	6878^b^ ^,^ ^e^ ^,^ ^f^

a Bisphenols are ordered by descending blood concentration.

b Highest concentration per compartment (among all bisphenols).

c Concentration predicted by the toddler model.

d Concentration predicted by the woman model.

e Concentration predicted by the child model.

f Concentration for which the steady state was not reached.

g Concentration predicted by the toddler and child model (with difference <1 pm).

**Figure 4:**
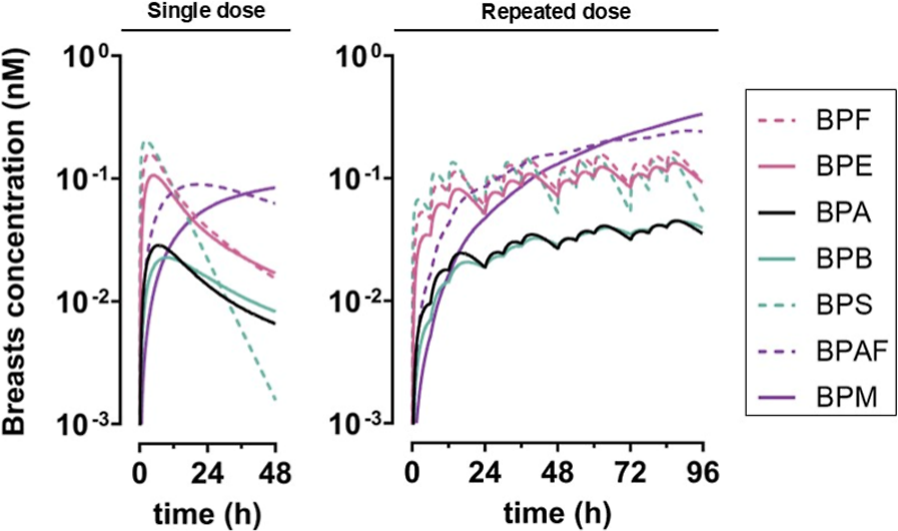
Predicted concentrations profiles (median of 10’000 MC simulations) of BPA, BPAF, BPB, BPE, BPF, BPM, and BPS in breasts after single dose exposure after single or repeated dose (exposure scenarios 6 and 10 of Table 1). Bisphenols are ordered by ascending MW.

The toxicokinetics of BPE, BPB, and BPM were modeled in humans for the first time and compared to other analogs. BPS, BPF, and BPE had the highest blood concentrations, were among the highest in tissues, and reached the steady state within 24–48 h (Figures 3, 4, and S8 and Table 3). BPE, with the third slowest glucuronidation rate and third lowest partition coefficients (Figure S3 and Table S5), consistently exhibited high concentrations across most organs (Figures 3, 4, and S8). In contrast, BPB had faster glucuronidation rates and higher blood–tissue partition coefficients (Figure S3 and Table S5), leading to its longer retention in the liver, where it was rapidly metabolized and excreted. BPB and BPA shared similar parameter values, resulting in lowest tissue concentrations, particularly in the thyroid, testes, and breasts, and steady-state achievement at ∼48 h (Figures 3, 4, and S8). More efficient glucuronidation rates in the current model predicted lower BPA concentrations compared to the previous model.^32^ BPM and BPAF are not structurally similar, but concentrations of both were among the lowest in blood and among the highest in thyroid and breasts (Figures 3, 4, and S8). While BPM underwent rapid hepatic glucuronidation (Figure S3), its kinetics differed significantly due to tissue/blood partitioning being several degrees of magnitude higher than that of other bisphenols. As a consequence, BPM concentration did not decrease during the 48 h following single dose exposure, did not reach the steady state in thyroid, testes, and breasts, and persisted beyond 96 h of simulation (Table 3 and Figures 3, 4, and S8).^22^

The present models predict bisphenol concentrations in three toxicologically relevant compartments related to evident toxic properties of BPA: thyroid, breasts (in women), and testes (in males). It is the first to predict concentrations of BPA analogs in the thyroid, for which their hormone-disrupting potential was being previously evaluated in vitro at much higher doses.^101^ Further, our model is the first to explore internal exposure to BPA alternatives in breasts, despite the carcinogenic potential of xenoestrogens in breast tissue. The potential effects of BPB, BPA, BPF, and BPS on estrogen receptor α, which can lead to tumor progression and metastasis, were previously studied in cell lines MCF-7, MDA-MB-231, and T47D cells at concentrations ranging from 10^–11^ to 10^–4^ M in the absence of precise exposure data.^102^ Concerningly, BPA was reported to exert increased combinatory estrogenicity in coexposure with other xenoestrogens.^6,103^ BPA analogs are commonly observed simultaneously in human biomonitoring.^16,18–20,22–25^ In this light, PBK models could serve as a starting point of selecting concentrations of BPA analogs to test for mixture effects and combinatory toxicity.

Compared to the previous model by Karrer et al.,^32^ these models more precisely parametrized male testes physiology^53^ and included three other bisphenols of potential reproductive toxicity. For instance, the impact of BPA, BPF, BPS, BPE, and BPB on the endocrine function of adult human testis explant cultures was tested at concentrations between 10^–9^ and 10^–5^ M.^104^ Cytotoxicity and phenotypic marker observations due to BPA, BPAF, BPF, BPS, and BPM exposure in germ and steroidogenic cell lines were also performed at concentrations ranging from 1 nM to 100 μM using single-cell high-content imaging.^105^ Our predictions provide a toxicokinetic perspective to these studies, clarifying their results and supporting further studies of BPA analog toxicity.

The presented PBK models predict significant differences in internal concentrations following oral exposure to equal levels of BPA and six analogs, highlighting the critical role of toxicokinetics in individual chemical toxicity. This underscores the need to avoid extrapolating BPA toxicity to structurally similar chemicals^106^ and emphasizes the urgency of conducting chemical-specific risk assessments for widely used BPA alternatives.

## Supplementary Material



## Notes

Examples of biomonitoring data indicating the importance of human exposure to bisphenol analogs; workflow of PBK model development, validation, reporting and dissemination; physiological parameters; physico-chemical properties of bisphenols included in the present study; physicochemical properties of bisphenol glucuronides; partition coefficients (P-) and unbound fractions (fu, FU-); chemicals, reagents, and enzymes used for glucuronidation kinetics; HPLC retention times of bisphenols; parameter variability estimates; estimated Papp across the Caco-2 monolayer determined by various techniques; predicted rates of glucuronidation of bisphenols in the human adult liver at physiologically relevant concentrations; hepatic glucuronidation kinetics; kinetics parameters for hepatic glucuronidation; EHC rate values; frequency of sensitivity of parameters across sensitivity analysis; Morris screening exercise for AUC of the blood concentration of BPA; Lowry plots of the eFAST quantitative measures for AUC of BPA blood concentration; mass balance analysis of excretion; predicted concentration profiles in woman (blood and thyroid) and toddler (blood, thyroid, and testes) models after single or repeated dose; and biomonitoring comparison (PDF)

## References

[1] Trullemans L., Koelewijn S. F., Scodeller I., Hendrickx T., Van Puyvelde P., Sels B. F. (2021). A guide towards safe, functional
and renewable BPA alternatives by rational molecular design: structure–property
and structure–toxicity relationships. Polym. Chem..

[2] EUROPEAN COMMISSION . Commission Directive 2011/8/EU of 28 January 2011 amending Directive 2002/72/EC as regards the restriction of use of Bisphenol A in plastic infant feeding bottles. 2011. https://eur-lex.europa.eu/legal-content/EN/TXT/HTML/?uri=CELEX:32011L0008 (accessed 09 09, 2025).

[3] EUROPEAN COMMISSION . Commission Regulation (EU) No 10/2011 of 14 January 2011 on plastic materials and articles intended to come into contact with food. 2011. https://eur-lex.europa.eu/legal-content/EN/TXT/PDF/?uri=OJ:L:2011:026:FULL (accessed 09 09, 2025).

[4] Canada Consumer Product Safety Act. M. o. Justice. 2012. https://laws-lois.justice.gc.ca/eng/acts/c-1.68/(accessed 09 09, 2025).

[5] FDA, U. S. Bisphenol A (BPA): Use in Food Contact Application. 2014. http://www.fda.gov/Food/IngredientsPackagingLabeling/FoodAdditivesIngredients/ucm064437.htm (accessed 09 09, 2025).

[6] Lee H., Park J., Park K. (2023). Mixture Effects
of Bisphenol A and
Its Structural Analogs on Estrogen Receptor Transcriptional Activation. Toxics.

[7] Marchiandi J., Alghamdi W., Dagnino S., Green M. P., Clarke B. O. (2024). Exposure
to endocrine disrupting chemicals from beverage packaging materials
and risk assessment for consumers. J. Hazard.
Mater..

[8] Wang R., Huang Y., Dong S., Wang P., Su X. (2021). The occurrence
of bisphenol compounds in animal feed plastic packaging and migration
into feed. Chemosphere.

[9] Cao L. Y., Ren X. M., Li C. H., Zhang J., Qin W. P., Yang Y., Wan B., Guo L. H. (2017). Bisphenol AF and
Bisphenol B Exert Higher Estrogenic Effects than Bisphenol A via G
Protein-Coupled Estrogen Receptor Pathway. Environ.
Sci. Technol..

[10] Della
Rocca Y., Traini E. M., Diomede F., Fonticoli L., Trubiani O., Paganelli A., Pizzicannella J., Marconi G. D. (2023). Current Evidence on Bisphenol A Exposure and the Molecular
Mechanism Involved in Related Pathological Conditions. Pharmaceutics.

[11] European Chemicals Agency . Agreement of the member state committee on the identification of 4,4’-(1-methylpropylidene)bisphenol (Bisphenol B) as a substance of very high concern, 2021. https://echa.europa.eu/documents/10162/dd9e01dd-8aac-8ecd-4c39-4da6c92c2ec8 (accessed 09 09, 2025).

[12] European Chemicals Agency . Substance Evaluation Conclusion and Evaluation Report for 4,4’-sulphonyldiphenol (Bisphenol S; BPS), 2022. https://echa.europa.eu/documents/10162/a4abf0c7-05f8-5139-74ff-fd287e295c93 (accessed 09 09, 2025).

[13] Escrivá L., Zilliacus J., Hessel E., Beronius A. (2021). Assessment of the endocrine
disrupting properties of bisphenol AF: a case study applying the European
regulatory criteria and guidance. Environ. Health.

[14] European Chemicals Agency . Bisphenols. https://www.echa.europa.eu/hot-topics/bisphenols (accessed 07 06, 24).

[15] Pallocca G., Moné M. J., Kamp H., Luijten M., van de Water B., Leist M. (2022). Next-generation risk assessment of chemicals – Rolling out
a human-centric testing strategy to drive 3R implementation: The RISK-HUNT3R
project perspective. Altex.

[16] Bommarito P. A., Stevens D. R., Welch B. M., Weller D., Meeker J. D., Cantonwine D. E., McElrath T. F., Ferguson K. K. (2023). Temporal
trends
and predictors of phthalate, phthalate replacement, and phenol biomarkers
in the LIFECODES Fetal Growth Study. Environ.
Int..

[17] Gálvez-Ontiveros Y., Moscoso-Ruiz I., Almazán Fernández de Bobadilla V., Monteagudo C., Giménez-Martínez R., Rodrigo L., Zafra-Gómez A., Rivas A. (2023). Levels of Bisphenol
A and its analogs in nails, saliva, and urine of children: a case
control study. Front. Nutr..

[18] Gys C., Bastiaensen M., Bruckers L., Colles A., Govarts E., Martin L. R., Verheyen V., Koppen G., Morrens B., Den Hond E., De Decker A., Schoeters G., Covaci A. (2021). Determinants of exposure
levels of bisphenols in flemish
adolescents. Environ. Res..

[19] Jeseta M., Kalina J., Franzova K., Fialkova S., Hosek J., Mekinova L., Crha I., Kempisty B., Ventruba P., Navratilova J. (2024). Cross sectional study on exposure to BPA and its analogues
and semen parameters in Czech men. Environ.
Pollut..

[20] Jiang V. S., Calafat A. M., Williams P. L., Chavarro J. E., Ford J. B., Souter I., Hauser R., Mínguez-Alarcón L. (2023). Temporal trends
in urinary concentrations of phenols, phthalate metabolites and phthalate
replacements between 2000 and 2017 in Boston, MA. Sci. Total Environ..

[21] Kolatorova
Sosvorova L., Chlupacova T., Vitku J., Vlk M., Heracek J., Starka L., Saman D., Simkova M., Hampl R. (2017). Determination of selected bisphenols, parabens and estrogens in human
plasma using LC-MS/MS. Talanta.

[22] Lucarini F., Krasniqi T., Bailat Rosset G., Roth N., Hopf N. B., Broillet M.-C., Staedler D. (2020). Exposure to
New Emerging Bisphenols
Among Young Children in Switzerland. Int. J.
Environ. Res. Public Health.

[23] Lyu Z., Harada K. H., Kim S., Fujitani T., Hitomi T., Pan R., Park N., Fujii Y., Kho Y., Choi K. (2023). Temporal trends
in bisphenol exposures and associated health risk among Japanese women
living in the Kyoto area from 1993 to 2016. Chemosphere.

[24] Xi J., Su X., Wang Z., Ji H., Chen Y., Liu X., Miao M., Liang H., Yuan W. (2023). The associations between
concentrations of gestational bisphenol analogues and thyroid related
hormones in cord blood: A prospective cohort study. Ecotoxicol. Environ. Saf..

[25] Zheng Q., Xiao J., Zhang D., Li X., Xu J., Ma J., Xiao Q., Fu J., Guo Z., Zhu Y., Ji J., Lu S. (2024). Bisphenol analogues
in infant foods in south China
and implications for infant exposure. Sci. Total
Environ..

[26] Dekant W., Völkel W. (2008). Human exposure
to bisphenol A by biomonitoring: methods,
results and assessment of environmental exposures. Toxicol. Appl. Pharmacol..

[27] Völkel W., Colnot T., Csanády G. A., Filser J. G., Dekant W. (2002). Metabolism
and Kinetics of Bisphenol A in Humans at Low Doses Following Oral
Administration. Chem. Res. Toxicol..

[28] Yang X., Doerge D. R., Teeguarden J. G., Fisher J. W. (2015). Development of a
physiologically based pharmacokinetic model for assessment of human
exposure to bisphenol A. Toxicol. Appl. Pharmacol..

[29] Gramec
Skledar D., Peterlin Mašič L. (2016). Bisphenol
A and its analogs: Do their metabolites have endocrine activity?. Environ. Toxicol. Pharmacol..

[30] Teeguarden J. G., Twaddle N. C., Churchwell M. I., Yang X., Fisher J. W., Seryak L. M., Doerge D. R. (2015). 24-h human
urine and serum profiles
of bisphenol A: Evidence against sublingual absorption following ingestion
in soup. Toxicol. Appl. Pharmacol..

[31] Oh J., Choi J. W., Ahn Y.-A., Kim S. (2018). Pharmacokinetics of
bisphenol S in humans after single oral administration. Environ. Int..

[32] Karrer C., Roiss T., von Goetz N., Gramec Skledar D., Peterlin Mašič L., Hungerbühler K. (2018). Physiologically
Based Pharmacokinetic (PBPK) Modeling of the Bisphenols BPA, BPS,
BPF, and BPAF with New Experimental Metabolic Parameters: Comparing
the Pharmacokinetic Behavior of BPA with Its Substitutes. Environ. Health Perspect..

[33] OECD . Guidance document on the characterisation, validation and reporting of Physiologically Based Kinetic (PBK) models for regulatory purposes, 2021. https://www.oecd.org/en/publications/guidance-document-on-the-characterisation-validation-and-reporting-of-physiologically-based-kinetic-pbk-models-for-regulatory-purposes_d0de241f-en.html (accessed 09 09, 2025).

[34] WHO . Joint FAO/WHO expert meeting to review toxicological and health aspects of bisphenol A: Final report, including report of stakeholder meeting on bisphenol A, 2011. http://apps.who.int/iris/bitstream/10665/44624/1/97892141564274_eng.pdf (accessed 09 09, 2025).

[35] EFSA
Panel on Food Contact Materials Enzymes Flavourings and Processing
Aids CEF (2015). Scientific opinion
on the risks to public health related to the presence of bisphenol
A (BPA) in foodstuffs. EFSA J..

[36] Brown R. P., Delp M. D., Lindstedt S. L., Rhomberg L. R., Beliles R. P. (1997). Physiological
parameter values for physiologically based pharmacokinetic models. Toxicol. Ind. Health.

[37] Davies B., Morris T. (1993). Physiological Parameters in Laboratory
Animals and
Humans. Pharm. Res..

[38] DeSesso J. M., Jacobson C. F. (2001). Anatomical and physiological parameters
affecting gastrointestinal
absorption in humans and rats. Food Chem. Toxicol..

[39] Helander H. F., Fändriks L. (2014). Surface area
of the digestive tract – revisited. Scand.
J. Gastroenterol..

[40] Valentin J. (2002). Basic anatomical
and physiological data for use in radiological protection: reference
values: ICRP Publication 89: Approved by the Commission in September
2001. Ann. ICRP.

[41] Levey A. S., Inker L. A., Coresh J. (2014). GFR estimation:
from physiology to
public health. Am. J. Kidney Dis..

[42] Meshkinpour H., Smith M., Hollander D. (1981). Influence
of aging on the surface
area of the small intestine in the rat. Exp.
Gerontol..

[43] Oatley K., Toates F. M. (1969). The passage of food
through the gut of rats and its
uptake of fluid. Psychon. Sci..

[44] Oberle R. L., Chen T.-S., Lloyd C., Barnett J. L., Owyang C., Meyer J., Amidon G. L. (1990). The influence
of the interdigestive
migrating myoelectric complex on the gastric emptying of liquids. Gastroenterology.

[45] Purdon R. A., Bass P. (1973). Gastric and Intestinal Transit in
Rats Measured by a Radioactive
Test Meal. Gastroenterology.

[46] Snipes R. L. (1997). Intestinal
absorptive surface in mammals of different sizes. Adv. Anat. Embryol. Cell Biol..

[47] Tanaka Y., Higashino H., Kataoka M., Yamashita S. (2020). Vivo Fluid
Volume in Rat Gastrointestinal Tract: Kinetic Analysis on the Luminal
Concentration of Nonabsorbable FITC-Dextran After Oral Administration. J. Pharm. Sci..

[48] Waidyanatha S., Black S. R., Aillon K., Collins B., Patel P. R., Riordan F., Sutherland V., Robinson V. G., Fernando R., Fennell T. R. (2019). Toxicokinetics and
bioavailability of bisphenol AF
following oral administration in rodents: A dose, species, and sex
comparison. Toxicol. Appl. Pharmacol..

[49] Willmann S., Höhn K., Edginton A., Sevestre M., Solodenko J., Weiss W., Lippert J., Schmitt W. (2007). Development of a Physiology-Based
Whole-Body Population Model for Assessing the Influence of Individual
Variability on the Pharmacokinetics of Drugs. J. Pharmacokinet. Pharmacodyn..

[50] Punt A., Pinckaers N., Peijnenburg A., Louisse J. (2021). Development of a Web-Based
Toolbox to Support Quantitative In-Vitro-to-In-Vivo Extrapolations
(QIVIVE) within Nonanimal Testing Strategies. Toxicol. Appl. Pharmacol..

[51] Rodgers T., Rowland M. (2006). Physiologically based
pharmacokinetic modelling 2:
Predicting the tissue distribution of acids, very weak bases, neutrals
and zwitterions. J. Pharm. Sci..

[52] Lobell M., Sivarajah V. (2003). In silico
prediction of aqueous solubility, human plasma
protein binding and volume of distribution of compounds from calculated
pKa and AlogP98 values. Mol. Divers..

[53] Pilari S., Gaub T., Block M., Görlitz L. (2017). Development
of Physiologically Based Organ Models to Evaluate the Pharmacokinetics
of Drugs in the Testes and the Thyroid Gland. CPT Pharmacometrics Syst. Pharmacol..

[54] Ulaszewska M. M., Ciffroy P., Tahraoui F., Zeman F. A., Capri E., Brochot C. (2012). Interpreting PCB levels in breast milk using a physiologically
based pharmacokinetic model to reconstruct the dynamic exposure of
Italian women. J. Expo. Sci. Environ. Epidemiol..

[55] Vandeweyer E., Hertens D. (2002). Quantification of glands
and fat in breast tissue:
An experimental determination. Toxicol Rep.

[56] Kamiya Y., Takaku H., Yamada R., Akase C., Abe Y., Sekiguchi Y., Murayama N., Shimizu M., Kitajima M., Shono F., Funatsu K., Yamazaki H. (2020). Determination and prediction
of permeability across intestinal epithelial cell monolayer of a diverse
range of industrial chemicals/drugs for estimation of oral absorption
as a putative marker of hepatotoxicity. Toxicol
Rep.

[57] Lanevskij K., Didziapetris R. (2019). Physicochemical
QSAR Analysis of Passive Permeability
Across Caco-2 Monolayers. J. Pharm. Sci..

[58] Chemaxon . LogD calculator. https://plugins.calculators.cxn.io/logd/(accessed 11 07, 2024).

[59] Zhao Y. H., Abraham M. H., Zissimos A. M. (2003). Determination
of McGowan Volumes
for Ions and Correlation with van der Waals Volumes. J. Chem. Inf. Comput..

[60] Sun D., Lennernas H., Welage L. S., Barnett J. L., Landowski C. P., Foster D., Fleisher D., Lee K.-D., Amidon G. L. (2002). Comparison
of Human Duodenum and Caco-2 Gene Expression Profiles for 12,000 Gene
Sequences Tags and Correlation with Permeability of 26 Drugs. Pharm. Res..

[61] Li C. Y., Basit A., Gupta A., Gáborik Z., Kis E., Prasad B. (2019). Major glucuronide metabolites of testosterone are primarily
transported by MRP2 and MRP3 in human liver, intestine and kidney. J. Steroid Biochem. Mol. Biol..

[62] Aichinger G., Stevanoska M., Beekmann K., Sturla S. J. (2023). Physiologically-Based
Pharmacokinetic Modeling of the Postbiotic Supplement Urolithin A
Predicts its Bioavailability Is Orders of Magnitude Lower than Concentrations
that Induce Toxicity, but also Neuroprotective Effects. Mol. Nutr. Food Res..

[63] Lee S., An K. S., Kim H. J., Noh H. J., Lee J., Lee J., Song K. S., Chae C., Ryu H. Y. (2022). Pharmacokinetics
and toxicity evaluation following oral exposure to bisphenol F. Arch. Toxicol..

[64] Yang X., Gandhi Y. A., Duignan D. B., Morris M. E. (2009). Prediction
of biliary
excretion in rats and humans using molecular weight and quantitative
structure-pharmacokinetic relationships. AAPS
J..

[65] Wang K., Jiang K., Wei X., Li Y., Wang T., Song Y. (2021). Physiologically Based Pharmacokinetic
Models Are Effective Support
for Pediatric Drug Development. AAPS PharmSciTech.

[66] Nalimov, V. V. The Application of Mathematical Statistics to Chemical Analysis; Pergamon Press, 1963.

[67] Wang X., He B., Shi J., Li Q., Zhu H.-J. (2020). Comparative Proteomics
Analysis of Human Liver Microsomes and S9 Fractions. Drug Metab. Dispos..

[68] Wang Q., Spenkelink B., Boonpawa R., Rietjens I., Beekmann K. (2020). Use of Physiologically
Based Kinetic Modeling to Predict Rat Gut Microbial Metabolism of
the Isoflavone Daidzein to S-Equol and Its Consequences for ERα
Activation. Mol. Nutr. Food Res..

[69] Barter E. Z., Bayliss K. M., Beaune H. P., Boobis R. A., Carlile J. D., Edwards J. R., Brian Houston J., Lake G. B., Lipscomb C. J., Pelkonen R. O., Tucke T. G., Rostami-Hodjegan A. (2007). Scaling Factors
for the Extrapolation of In Vivo Metabolic Drug Clearance From In
Vitro Data: Reaching a Consensus on Values of Human Micro-somal Protein
and Hepatocellularity Per Gram of Liver. Curr.
Drug Metab..

[70] Deepika D., Sharma R. P., Schuhmacher M., Sakhi A. K., Thomsen C., Chatzi L., Vafeiadi M., Quentin J., Slama R., Grazuleviciene R., Andrušaitytė S., Waiblinger D., Wright J., Yang T. C., Urquiza J., Vrijheid M., Casas M., Domingo J. L., Kumar V. (2022). Unravelling sex-specific
BPA toxicokinetics in children using a pediatric PBPK model. Environ. Res..

[71] Bhatt D. K., Mehrotra A., Gaedigk A., Chapa R., Basit A., Zhang H., Choudhari P., Boberg M., Pearce R. E., Gaedigk R., Broeckel U., Leeder J. S., Prasad B. (2019). Age- and Genotype-Dependent
Variability in the Protein Abundance and Activity of Six Major Uridine
Diphosphate-Glucuronosyltransferases in Human Liver. Clin. Pharmacol. Ther..

[72] Thayer K. A., Doerge D. R., Hunt D., Schurman S. H., Twaddle N. C., Churchwell M. I., Garantziotis S., Kissling G. E., Easterling M. R., Bucher J. R., Birnbaum L. S. (2015). Pharmacokinetics of bisphenol A in
humans following a single oral administration. Environ. Int..

[73] Derendorf, H. ; Schmidt, S. Rowland and Tozer’s Clinical Pharmacokinetics and Pharmacodynamics: Concepts and Applications, 5e; Lippincott Williams & Wilkins, a Wolters Kluwer business, 2020.

[74] Evans M. V., Andersen M. E. (2000). Sensitivity analysis of a physiological
model for 2,3,7,8-tetrachlorodibenzo-p-dioxin
(TCDD): assessing the impact of specific model parameters on sequestration
in liver and fat in the rat. Toxicol. Sci..

[75] McNally K., Cotton R., Loizou G. D. (2011). A Workflow
for Global Sensitivity
Analysis of PBPK Models. Front. Pharmacol.

[76] Herman J., Usher W. (2017). SALib An open-source Python library
for Sensitivity Analysis. J. Open Source Softw..

[77] Clewell H. J., Gearhart J. M., Gentry P. R., Covington T. R., VanLandingham C. B., Crump K. S., Shipp A. M. (1999). Evaluation of the
uncertainty in an oral reference dose for methylmercury due to interindividual
variability in pharmacokinetics. Risk Anal..

[78] Clewell R. A., Clewell H. J. (2008). Development and
specification of physiologically based
pharmacokinetic models for use in risk assessment. Regul. Toxicol. Pharmacol..

[79] Fravel M. A., Ernst M. E., Webb K. L., Wetmore J. B., Wolfe R., Woods R. L., Reid C. M., Chowdhury E., Murray A. M., Polkinghorne K. R. (2023). GFR Variability, Survival, and Cardiovascular
Events in Older Adults. Kid Med..

[80] Guiastrennec B., Sonne D. P., Bergstrand M., Vilsbøll T., Knop F. K., Karlsson M. O. (2018). Model-Based Prediction
of Plasma
Concentration and Enterohepatic Circulation of Total Bile Acids in
Humans. CPT Pharmacometrics Syst. Pharmacol..

[81] Thomas R. S., Lytle W. E., Keefe T. J., Constan A. A., Yang R. S. H. (1996). Incorporating
Monte Carlo Simulation into Physiologically Based Pharmacokinetic
Models Using Advanced Continuous Simulation Language (ACSL): A Computational
Method. Fundam. Appl. Toxicol..

[82] Ahire D., Patel M., Deshmukh S. V., Prasad B. (2023). Quantification of Accurate
Composition and Total Abundance of Homologous Proteins by Conserved-Plus-Surrogate
Peptide Approach: Quantification of UDP Glucuronosyltransferases in
Human Tissues. Drug Metab. Dispos..

[83] Audebert M., Dolo L., Perdu E., Cravedi J. P., Zalko D. (2011). Use of the
γH2AX assay for assessing the genotoxicity of bisphenol A and
bisphenol F in human cell lines. Arch. Toxicol..

[84] Mazur C. S., Kenneke J. F., Hess-Wilson J. K., Lipscomb J. C. (2010). Differences between
Human and Rat Intestinal and Hepatic Bisphenol A Glucuronidation and
the Influence of Alamethicin on In Vitro Kinetic Measurements. Drug Metab. Dispos..

[85] Trdan
Lušin T., Roškar R., Mrhar A. (2012). Evaluation of bisphenol
A glucuronidation according to UGT1A1*28 polymorphism by a new LC–MS/MS
assay. Toxicology.

[86] Durcik M., Gramec Skledar D., Tomašič T., Trontelj J., Peterlin
Mašič L. (2022). Last piece in the puzzle of bisphenols
BPA, BPS and BPF metabolism: Kinetics of the in vitro sulfation reaction. Chemosphere.

[87] Hill C. E., Sapouckey S. A., Suvorov A., Vandenberg L. N. (2017). Developmental
exposures to bisphenol S, a BPA replacement, alter estrogen-responsiveness
of the female reproductive tract: A pilot study. Cogent Med..

[88] Wu X., Yang X., Tian Y., Xu P., Yue H., Sang N. (2023). Bisphenol B and bisphenol AF exposure
enhances uterine diseases risks
in mouse. Environ. Int..

[89] Demierre A.-L., Peter R., Oberli A., Bourqui-Pittet M. (2012). Dermal penetration
of bisphenol A in human skin contributes marginally to total exposure. Toxicol. Lett..

[90] Hu M., Zhang Z., Zhang Y., Zhan M., Qu W., He G., Zhou Y. (2023). Development of human dermal PBPK models for the bisphenols
BPA, BPS, BPF, and BPAF with parallel-layered skin compartment: Basing
on dermal administration studies in humans. Sci. Total Environ..

[91] von
Goetz N., Pirow R., Hart A., Bradley E., Poças F., Arcella D., Lillegard I. T. L., Simoneau C., van Engelen J., Husoy T., Theobald A., Leclercq C. (2017). Including non-dietary sources into an exposure assessment
of the European Food Safety Authority: The challenge of multi-sector
chemicals such as Bisphenol A. Regul. Toxicol.
Pharmacol..

[92] Mazur C. S., Marchitti S. A., Dimova M., Kenneke J. F., Lumen A., Fisher J. (2012). Human and
Rat ABC Transporter Efflux of Bisphenol A
and Bisphenol A Glucuronide: Interspecies Comparison and Implications
for Pharmacokinetic Assessment. Toxicol. Sci..

[93] Artacho-Cordón F., Arrebola J. P., Nielsen O., Hernández P., Skakkebaek N. E., Fernández M. F., Andersson A. M., Olea N., Frederiksen H. (2017). Assumed non-persistent
environmental
chemicals in human adipose tissue; matrix stability and correlation
with levels measured in urine and serum. Environ.
Res..

[94] Fernandez M. F., Arrebola J. P., Taoufiki J., Navalón A., Ballesteros O., Pulgar R., Vilchez J. L., Olea N. (2007). Bisphenol-A
and chlorinated derivatives in adipose tissue of women. Reprod. Toxicol..

[95] Geens T., Neels H., Covaci A. (2012). Distribution
of bisphenol-A, triclosan
and n-nonylphenol in human adipose tissue, liver and brain. Chemosphere.

[96] Keshavarz-Maleki R., Kaviani A., Omranipour R., Gholami M., Khoshayand M. R., Ostad S. N., Sabzevari O. (2021). Bisphenol-A
in biological samples
of breast cancer mastectomy and mammoplasty patients and correlation
with levels measured in urine and tissue. Sci.
Rep..

[97] Liu Y., Yan Z., Zhang Q., Song N., Cheng J., Torres O. L., Chen J., Zhang S., Guo R. (2019). Urinary levels, composition
profile and cumulative risk of bisphenols in preschool-aged children
from Nanjing suburb, China. Ecotoxicol. Environ.
Saf..

[98] Wang L., Asimakopoulos A. G., Kannan K. (2015). Accumulation of 19 environmental
phenolic and xenobiotic heterocyclic aromatic compounds in human adipose
tissue. Environ. Int..

[99] Zhou X., Kramer J. P., Calafat A. M., Ye X. (2014). Automated
on-line column-switching
high performance liquid chromatography isotope dilution tandem mass
spectrometry method for the quantification of bisphenol A, bisphenol
F, bisphenol S, and 11 other phenols in urine. J. Chromatogr. B.

[100] Golosovskaia E., Örn S., Leonards P., Koekkoek J., Andersson P. L. (2025). Studying
interaction effects on toxicokinetics in zebrafish
combining experimental and modelling approaches. Sci. Total Environ..

[101] Wu X., Yang X., Geng X., Ji X., Zhang X., Yue H., Li G., Sang N. (2022). Bisphenol
A Analogs Induce Cellular
Dysfunction in Human Trophoblast Cells in a Thyroid Hormone Receptor-Dependent
Manner: In Silico and In Vitro Analyses. Environ.
Sci. Technol..

[102] Mesnage R., Phedonos A., Arno M., Balu S., Corton J. C., Antoniou M. N. (2017). Editor’s Highlight: Transcriptome
Profiling Reveals Bisphenol A Alternatives Activate Estrogen Receptor
Alpha in Human Breast Cancer Cells. Toxicol.
Sci..

[103] Aichinger G., Pantazi F., Marko D. (2020). Combinatory
estrogenic
effects of bisphenol A in mixtures with alternariol and zearalenone
in human endometrial cells. Toxicol. Lett..

[104] Desdoits-Lethimonier C., Lesné L., Gaudriault P., Zalko D., Antignac J. P., Deceuninck Y., Platel C., Dejucq-Rainsford N., Mazaud-Guittot S., Jégou B. (2017). Parallel assessment of the effects of bisphenol A and
several of its analogs on the adult human testis. Hum. Reprod..

[105] Rajkumar A., Luu T., Beal M. A., Barton-Maclaren T. S., Robaire B., Hales B. F. (2021). Elucidation
of the Effects of Bisphenol
A and Structural Analogs on Germ and Steroidogenic Cells Using Single
Cell High-Content Imaging. Toxicol. Sci..

[106] Mhaouty-Kodja S., Zalko D., Tait S., Testai E., Viguié C., Corsini E., Grova N., Buratti F. M., Cabaton N. J., Coppola L., De la Vieja A., Dusinska M., El Yamani N., Galbiati V., Iglesias-Hernández P., Kohl Y., Maddalon A., Marcon F., Naulé L., Rundén-Pran E., Salani F., Santori N., Torres-Ruiz M., Turner J. D., Adamovsky O., Aiello-Holden K., Dirven H., Louro H., Silva M. J. (2024). A critical review
to identify data gaps and improve risk assessment of bisphenol A alternatives
for human health. Crit. Rev. Toxicol..

