# Supplementary material for: Human Internal Exposures of Bisphenol A and Six Data-Poor Analogs Predicted by Physiologically Based Kinetic Models with Multimodal Parametrization
Source: Environ Sci Technol. 2025 Sep 25;59(39):20919–30. doi: 10.1021/acs.est.5c00513 (PMC12509328; doi:10.1021/acs.est.5c00513)
Supplement: Supplementary file 1 [file es5c00513_si_001.pdf]

Supporting information

Human internal exposures of bisphenol A and six data-poor analogs predicted by physiologically based kinetic models with multimodal parameterization

*Hélène Bigonne, Amrei Rolof, Inga Potapova, Shana J. Sturla and Georg Aichinger**

Department of Health Sciences and Technology, Institute of Food Nutrition and Health, ETH Zürich, CH-8092 Zürich, Switzerland.

*georg.aichinger@hest.ethz.ch

Summary:

- Number of pages: 42
- Number of tables: 12
- Number of figures: 8

Table of Contents

Table S1: Examples of biomonitoring data indicating the importance of human exposure to bisphenols analogs 3

Figure S1: Workflow of PBK model development, validation, reporting and dissemination 4

Table S2: Physiological parameters. 5

Supplementary information on physiological parameters 6

Table S3: Physico-chemical properties of bisphenols included in the present study. 8

Table S4: Physicochemical properties of bisphenol glucuronides. 9

Table S5: Partition coefficients (P-) and unbound fractions (fu, FU-). 10

Supplementary information on glucuronidation kinetics measurement 11

Table S6: Chemicals, Reagents and Enzymes used for glucuronidation kinetics 12

Table S7: HPLC retention times of bisphenols. 13

Table S8: Parameter Variability Estimates 14

Figure S2. Estimated Papp across the Caco-2 monolayer determined by various techniques 17

Figure S3. Predicted rates of glucuronidation of bisphenols in human adult liver at physiologically relevant concentrations. 18

Figure S4: Hepatic glucuronidation kinetics. 19

Table S9: Kinetics parameters for hepatic glucuronidation. 20

Table S10: EHC rate values. 20

Figure S5: Frequency of sensitivity of parameters across sensitivity analysis. 21

Figure S6: Morris screening exercise 22

Figure S7: Lowry plots of the eFAST quantitative measures 23

Table S11: Mass balance analysis of excretion 24

Figure S8: Predicted concentration profiles in woman (blood, thyroid) and toddler (blood, thyroid, testes) models after single or repeated dose. 25

Supplementary information on biomonitoring comparison 26

Table S12: Biomonitoring comparison 27

Model code (Berkeley Madonna) 31

References 39

Table S1: Examples of biomonitoring data indicating the importance of human exposure to bisphenols analogs

Location	Population	Matrix	Importance of exposure to BPA analog(s)	Reference
US	pregnant women	urine	biomarkers of **BPS** increased from 2007 to 2018	Bommarito et al. (2023)
US	men and women	urine	BPA levels declined alongside a rise in **BPF** and **BPS** levels between 2000 and 2017	Jiang et al. (2023)
Japan	healthy women	urine	BPA levels declined alongside a rise in **BPF** and **BPE** levels between 1993 and 2016	Lyu et al. (2023)
Czech Republic	healthy normospermic men	plasma	**BPF** levels were three times higher than BPA levels.	Kolatorova Sosvorova et al. (2017)
south China	breastfeeding women	breastmilk	**BPS** was the most frequently detected among BPA and eight analogs (53.2 % of samples)	Zheng et al. (2024)
Czech Republic	men	semen	**BPS** was detected in over 80% of samples	Jeseta et al. (2024)
China	pregnant women	urine	**BPAF** was detected in 28% of samples	Xi et al. (2023)
Belgium	male and female adolescents	urine	**BPB** was the 4th most prevalent bisphenol<br>BPA levels decreased significantly from 2008 to 2018	Gys et al. (2021)
Switzerland	infants and toddlers aged between 6 and 36 months	urine	**BPM** was detected with the highest concentration (8.56 µg/g) among BPA and 14 emerging BP analogues	Lucarini et al. (2020)
Spain	obese and non-obese children between 6 and 12 years old	saliva	**BPM** was frequently detected	Gálvez-Ontiveros et al. (2023)

Figure S1: Workflow of PBK model development, validation, reporting and dissemination (adapted from OECD (2021)).

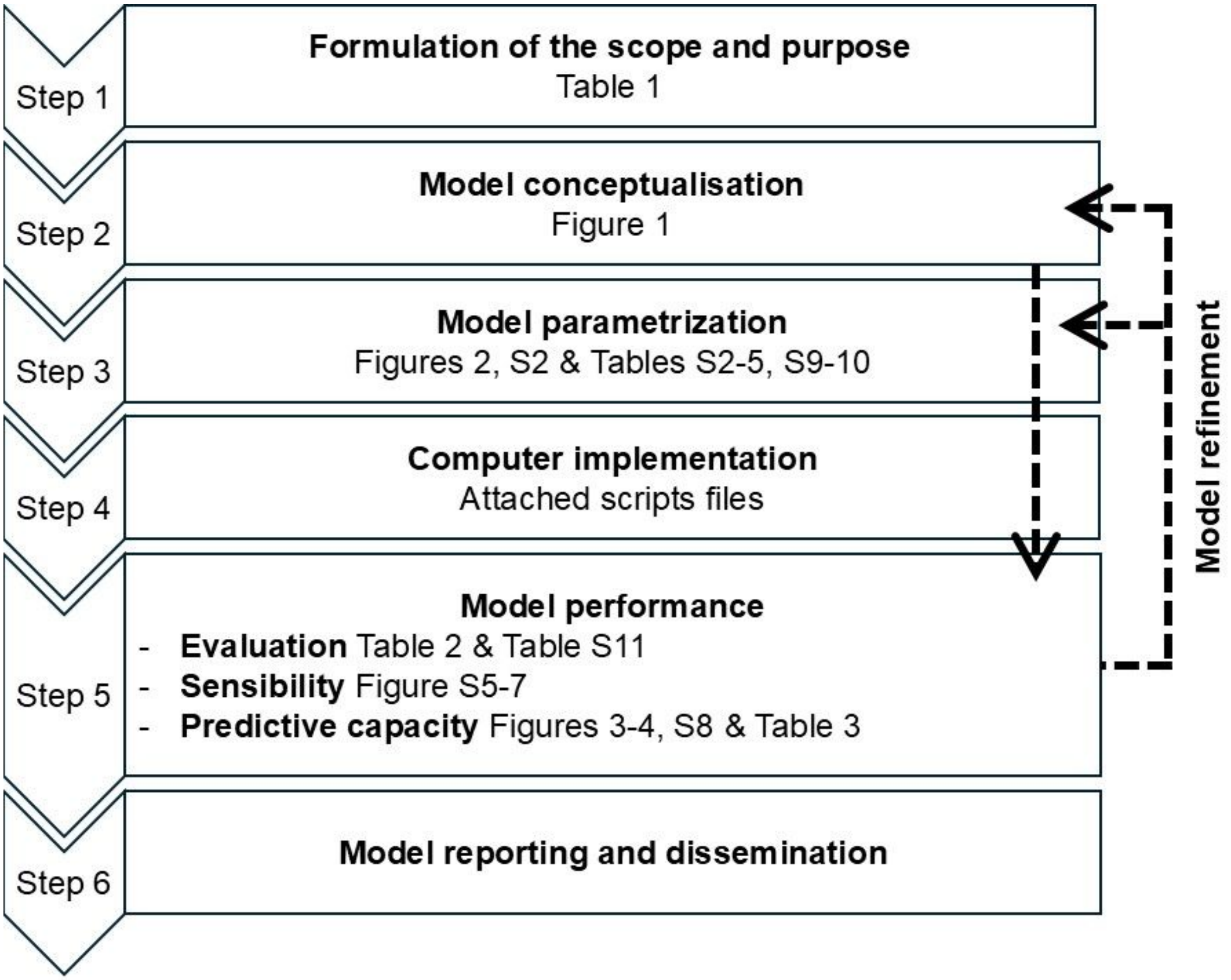

Table S2: Physiological parameters.

	MAN	WOMAN	CHILD	TODDLER	RAT
Body weight (BW, kg)	73	60	19	10	0.25
Height (H, cm)	176	163	109	76	-
Age	30 years	30 years	5 years	1 year	11 weeks

- ***Fractional organ volumes (% of body weight)***

VLC - Liver volume	3.19	2.98	3.74	3.80	3.4
VFC - Adipose volume	20.36	30.55	19.32	23.43	7
VSKC - Skin volume	4.74	4.03	3.22	3.65	19.7
VBRC - Brain volume	2.07	2.24	6.98	9.56	0.6
VSTC- Stomach volume	0.28	0.30	0.34	0.25	0.52
VTSC- Testes volume	0.05	-	0.01	0.02	-
VBSC - Breasts volume	-	0.83	-	-	-
VTHC - Thyroid volume	0.03	0.03	0.02	0.02	-
VBC - Blood volume	7.26	6.50	7.37	5.00	7.4
VRC - Rapidly perfused tissues volume	2.69	2.58	2.83	2.78	1.51
VSLC - Slowly perfused tissues volume	55.63	43.30	43.81	31.58	47.7
VGLC - Gut lumen volume	0.89	1.00	1.14	1.73	0.9
VGTC - Gut tissue volume	1.40	1.60	1.79	1.35	1.62
VKC – Kidneys volume	0.6	0.6	0.7	0.8	0.73

- ***Intestinal surface areas (SA) (dm2)***

SASI small intestine	2917.5	2709.1	1771.3	1250.3	-
SALI large intestine	107.8	107.8	73.5	58.8	-
ISA - total intestinal SA (dm2)	3025.3	2816.9	1844.8	1309.1	1.44

- ***Further physiological parameters***

TRANSI - transit time (h)	40	52	38	36	18
GEst - time to empty 50% of stomach (min)	11.8	a	a	a	25
GFR (ml/min [/1.73 m2 for human])	125	a	a	a	1.31
CO - Cardiac output (L/min)	6.5	5.9	3.4	1.2	0.083

- ***Fractional blood flows (% or cardiac output)***

QLC - Liver debit	6.5	6.5	b	b	18.3
QFC - Adipose debit	5	8.5	b	b	7.0
QRC - Rapidly perfused tissues debit	123	122	b	b	21.3
QSLC - Slowly perfused tissues debit	22	17	b	b	40
QSKC - Skin debit	5	5	b	b	5.8
QGTC - Gut tissue debit	14	16	b	b	9.0
QBRC - Brain debit	12	12	b	b	2.0
QTHC - Thyroid debit	1.5	1.5	b	b	-
QTSC - Testes debit	0.05	-	b	b	-
QBSC - Breasts debit	-	0.4	-	-	-
QKC – Kidneys debit	19	17	b	b	14.11

a: Value conserved for all human models
b: Value conserved for all male models

Supplementary information on physiological parameters

Human physiological parameters were predominantly sourced from the International Commission on Radiological Protection (ICRP) report 89. (ICRP, 2002) Fractional organ volumes were computed with the formula (1), except for breasts volume, which was the ratio between breasts and whole-body weight.

$$Fractional\ organ\ volume\ = \frac{organ\ mass\ without\ blood\ (g) + (\ \frac{regional\ blood\ flow\ (\%)}{100} \times body\ blood\ volume\ (mL)\ )}{body\ weight\ (g)} \quad [1]$$

The volumes of combined compartments were the sum of their constituent organ volumes. Concerning gut tissue fractional volume, the total mass of gastrointestinal tract walls (excluding esophagus and stomach) was divided by body weight, while the fractional volume of gut lumen was estimated by dividing the total mass of gastrointestinal tract content (excluding the stomach) by body weight. As described by Helander and Fändriks (2014), total intestinal surface area (ISA) was modeled by approximating the small intestine (SI) and large intestinal (LI) as cylinders enlarged with folds, villi and microvilli. (Helander & Fändriks, 2014; Snipes, 1997) Length values for SI and LI were adjusted based on age and sex, while diameter values from Helander and Fändriks (2014) were the same for all human models. Blood flow rates (as % of cardiac output) were set from adult values. Lung blood flow rate issued from Willmann et al. (2007). The gut tissue blood flow rate was the sum of SI and LI blood flow rate. The blood flow rates of combined compartments were the sum of their constituent organs blood flow rates. Gastric emptying half-time was measured in Oberle et al. (1990) for an administered volume of 200 mL, to approach the conditions of the toxicokinetic study. (Teeguarden et al., 2015) The intestinal transit time was defined as the sum of the transit times of luminal contents through SI, right colon, left colon, and sigmoid colon. Human glomerular filtration rate (GFR) was sourced from Levey et al. (2014)

Rat physiological parameters were primarily sourced from Brown et al. (1997) to represent an eleven-week-old Sprague Dawley rat weighing 250g, with no specification of sex. Stomach and gut tissue (intestine) volume were measured by Oatley and Toates (1969) in hooded rats. Gut lumen volume was the sum of fluid volumes in the duodenum, upper jejunum, lower jejunum and ileum for Wistar rats following oral administration of 1 mL. (Tanaka et al., 2020) Intestinal surface area value was sourced from Meshkinpour et al. (1981). Gut tissue blood flow rate (as % of cardiac output) was sourced by Davies and Morris (1993). The blood flow rates of combined compartments were the sum of their constituent organs blood flow rates. The gastric emptying half-time for a volume of 1 mL was extracted from Purdon and Bass (1973) to approach the volume delivered in animal study (1.25 mL, Waidyanatha et al. (2019)). Intestinal transit time was the sum of the values reported for chyme transit times through SI and LI. (DeSesso & Jacobson, 2001) The rat GFR value was sourced from Davies and Morris (1993).

Table S3: Physico-chemical properties of bisphenols included in the present study.

Bisphenol	Structure	MW[a]	*pKa*[b]	*pKa*[c]	**pKa[d]**	*logP*[b]	*logP*[c]	**logP[d]**	CAS
BPF	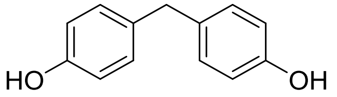	200.24	*9.66*	*9.46*	**9.56**	*3.35*	*3.45*	**3.30**	620-92-8
BPE	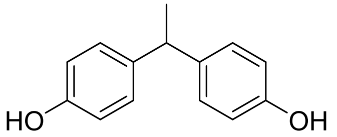	214.26	*9.59*	*9.81*	**9.70**	*3.68*	*3.74*	**3.71**	2081-08-5
BPA	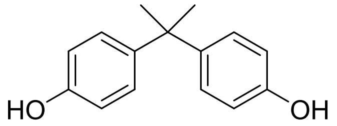	228.12	*9.52*	*9.78*	**9.65**	*4.15*	*4.04*	**4.10**	80-05-7
BPB	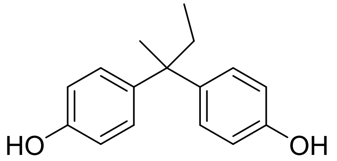	242.32	*9.49*	*9.77*	**9.63**	*4.57*	*4.49*	**4.53**	77-40-7
BPS	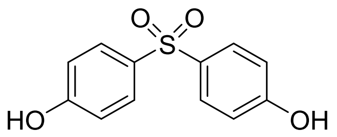	250.27	*8.44*	*7.42*	**7.93**	*2.15*	*2.32*	**2.24**	80-09-1
BPAF	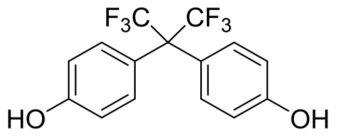	336.06	*8.90*	*9.75*	**9.33**	*5.02*	*4.75*	**4.89**	1478-61-1
BPM	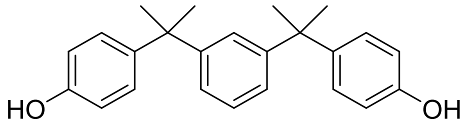	346.47	*9.47*	*9.78*	**9.63**	*7.04*	*6.72*	**6.88**	13595-25-0

[a] Values given in g/mol. Bisphenols are ordered by ascending MW ; [b] Parameter predicted by ChemDraw 20.0 ; [c] Parameter predicted by Chemaxon Playground v1.6.2 ; [d] Average parameter prediction used in the model.

Table S4: Physicochemical properties of bisphenol glucuronides.

	MW (g/mol)	*pKa*[a]	*pKa*[b]	**pKa**[c]	*logP*[a]	*logP*[b]	**logP**[c]
BPA glucuronide	404.42	*3.26*	*3.25*	**3.26**	*2.27*	*2.10*	**2.19**
BPAF glucuronide	512.36	*3.26*	*3.14*	**3.20**	*3.13*	*2.83*	**2.98**
BPB glucuronide	418.44	*3.25*	*3.25*	**3.25**	*2.68*	*2.54*	**2.61**
BPE glucuronide	390.39	*3.26*	*3.26*	**3.26**	*1.79*	*1.80*	**1.80**
BPF glucuronide	376.36	*3.26*	*3.27*	**3.27**	*1.46*	*1.51*	**1.49**
BPM glucuronide	522.59	*3.25*	*3.25*	**3.25**	*5.16*	*4.78*	**4.97**
BPS glucuronide	426.39	*3.25*	*4.20*	**3.73**	*0.26*	*0.37*	**0.32**

[a] Parameter predicted by ChemDraw 20.0 ; [b] Parameter predicted by Chemaxon Playground v1.6.2 ; [c] Average parameter prediction used in the model.

Table S5: Partition coefficients (P-) and unbound fractions (fu, FU-).

	BPF	BPE	BPA	BPB	BPS	BPAF	BPM
Adipose tissue (PFBP)	16.33	31.7	58.75	115.26	1.28	199.61	4351.95
Brain (PBRBP)	7.19	12.41	20.69	36.19	1.06	57.27	732.35
Breasts (PBSBP)	4.00	7.77	14.39	28.24	0.31	48.90	1066.23
Gut tissue (PGTBP)	7.53	12.94	21.52	37.57	1.18	59.39	758.23
Liver (parent, PLBP)	3.8	6.5	10.79	18.81	0.64	29.72	379.34
Liver (glucuronide, PLBPgluc)	0.11	0.11	0.1	0.1	0.51	0.09	7.29
Rapidly perfused (PRBP)	4.13	7.02	11.59	20.16	0.74	31.81	404.95
Lungs	*4.67*	*7.93*	*13.11*	*22.8*	*0.83*	*35.98*	*457.97*
Kidneys	*3.58*	*6.09*	*10.07*	*17.52*	*0.65*	*27.66*	*352.4*
Heart	*3.12*	*5.27*	*8.68*	*15.08*	*0.59*	*23.78*	*302.47*
Skin (PSKBP)	11.24	19.33	32.14	56.09	1.73	88.66	1131.57
Slowly perfused (PSLBP)	2.55	4.32	7.16	12.46	0.47	19.68	250.89
Muscles	*2.28*	*3.85*	*6.36*	*11.07*	*0.44*	*17.48*	*222.87*
Bones	*3.29*	*5.62*	*9.33*	*16.25*	*0.55*	*25.68*	*327.43*
Testes (PTSBP)	2.30	2.2	3.07	4.51	2.56	11.42	426.24
Thyroid (PTHBP)	2.49	3.10	5.42	10.50	3.83	39.13	1773.52
fu (parent, FUBP)	0.091	0.061	0.042	0.027	0.17	0.019	0.002
fu (glucuronide, FUBPG)	0.099	0.078	0.057	0.041	0.227	0.03	0.006

Supplementary information on glucuronidation kinetics measurement

BPA, BPB, BPE and BPM were incubated with Corning® UltraPool™ Human Liver S9 in a buffer containing UDPGA. The S9 reagent (20 mg protein/mL) was aliquoted and stored at -80° C until use. BPAF and BPF were incubated similarly with rat liver S9 (Sprague-Dawley, male, 0.1 mg/mL S9 protein). For each assay, an S9 aliquot was thawed on ice, and a reaction master mix was prepared with 0.1 mg S9 protein/mL in either 0.1 M (for BPA, BPB, BPAF, and BPF) or 0.25 mg/mL (for BPE and BPM) Tris-HCl, along with 10 mM UDPGA in 50 mM Tris-HCl (pH 7.4), 25 µg/mL alamethicin, and 10 mM magnesium chloride. Negative controls using 0.1 M Tris-HCl without UDPGA were included. Each reaction solution (100 µL) was pre-incubated for 5 min at 37° C with 350 rpm shaking. To this solution was added bisphenol solutions (0 to 20 mM) in DMSO in a ratio of 1:100 to achieve final bisphenol concentrations of 0 to 200 µM and 1% (v/v) DMSO. The shortest incubation times for maximal reaction speeds were identified: 10 min for BPE, 15 min for BPB, 20 min for BPA and BPM, 60 min for BPF, and 120 min for BPAF. The glucuronidation reaction was stopped by adding cold methanol (160 µL) and samples were kept at -20 °C for 1 h to precipitate proteins. The resulting mixture was centrifuged (10 min, 4 °C, 15,000 rcf). Supernatants were analyzed using High-performance liquid chromatography (HPLC) with diode array detector (DAD). Similar experiments were conducted for BPA, BPB, BPE, and BPM with human small intestinal S9 mix (0.025 to 0.25 mg/mL). Bisphenol concentrations ranged from 5 to 120 µM, and incubation times from 15 to 120 min.

The quantification of the bisphenols and their glucuronides was carried out on an Agilent 1200 series HPLC system with a Waters XBridge BEH130 C18 column (4.6 × 150 mm, 3.5 µm particle size) for separation and a DAD set to a detection wavelength of λ = 280 nm. A gradient elution was used with water containing 0.1% formic acid as eluent A and acetonitrile with 0.1% formic acid as eluent B. The initial condition of 15% eluent B was increased to 65% eluent B over 20 min, and then to 90% eluent B in the next 5 min. The retention times for the various bisphenols and their glucuronides are provided in Supplementary Table S7. Calibration curves were set from concentrations ranging from 1 to 100 µM.

Table S6: Chemicals, Reagents and Enzymes used for glucuronidation kinetics

Material	Supplier
Corning UltraPool Human Liver S9 (mixed gender, 150-donorpool)	Corning
Corning Gentest UGT Reaction Mix	Corning
Pooled human intestinal S9 fraction	Biopredic
Uridine-5'-diphosphoglucuronic acid (UDPGA) trisodium salt	Sigma–Aldrich
Magnesiumchloride	Sigma–Aldrich
ß-Glucuronidases (from bovine liver)	Sigma–Aldrich
Sodium acetate	Sigma–Aldrich
Alamethicin	Enzo Life Sciences AG
Urolithin A (3,8-Dihydroxy-6H-benzo[c]chromen-6-one)	abcr
Bisphenol A	Sigma–Aldrich
Bisphenol AF	Sigma–Aldrich
Bisphenol B	Sigma–Aldrich
Bisphenol E	Sigma–Aldrich
Bisphenol F	Sigma–Aldrich
Bisphenol M	Sigma–Aldrich
Bisphenol S	Sigma–Aldrich
DMSO	VWR
Acetonitrile (ACN, HPLC-grade)	Merck-Millipore
Tris–HCl	Fluka Chemikals
Rat liver S9 (Sprague-Dawley, male)	Sigma-Aldrich
Formic acid	Fischer scientific

Table S7: HPLC retention times of bisphenols.

Bisphenol	Retention time tr (min)	
	Parent	Metabolite
BPA	14.8	10.1
BPAF	18.1	13.1
BPB	16.6	11.4
BPE	13.5	9.1
BPF	12.0	7.8
BPM	22.4	17

Table S8: Parameter Variability Estimates

Parameter		Sc > 0.1	CV (%)	Distribution	Reference
BW	Body weight (kg)	yes	26	Lognormal	(Clewell et al., 1999)
H	Height (cm)	no	10	Lognormal	(McNally et al., 2011)
SASI	Surface area small intestine (dm2)	no	30	Lognormal	estimated
SALI	Surface area large intestine (dm2)	no	30	Lognormal	estimated
• Organ volumes (fraction of BW)					
VL	Liver volume	yes	25	Normal	(Clewell et al., 1999)
VR	Rapidly perfused tissues volume	yes	30	Normal	(Clewell & Clewell, 2008)
VSL	Slowly perfused tissues volume	yes	16	Normal	(Clewell et al., 1999)
VTS	Testes volume	yes	30	Normal	(Clewell & Clewell, 2008)
VBR	Brain volume	yes	30	Normal	(Clewell & Clewell, 2008)
VF	Adipose volume	yes	24	Normal	(Clewell et al., 1999)
VTH	Thyroid volume	yes	30	Normal	(Clewell & Clewell, 2008)
VBS	Breasts volume	yes	30	Normal	(Clewell & Clewell, 2008)
VSK	Skin volume	no	30	Normal	(Clewell & Clewell, 2008)
VST	Stomach volume	no	30	Normal	(Clewell & Clewell, 2008)
VK	Kidneys volume	no	30	Normal	(Clewell & Clewell, 2008)
VP	Plasma volume	no	30	Normal	(Clewell & Clewell, 2008)
VGL	Gut lumen volume	no	30	Normal	(Clewell & Clewell, 2008)
VGT	Gut tissue volume	no	30	Normal	(Clewell & Clewell, 2008)
• Further physiological parameters					
CO	Cardiac output (L/min)	yes	22	Normal	(Clewell et al., 1999)

TRANSI	Transit time (h)	no	22	Lognormal	estimated
EHCr	EHC rate	yes	0.59	Lognormal	(Guiastrennec et al., 2018)
GEst	Time to empty 50% of stomach (min)	yes	69	Lognormal	(Oberle et al., 1990)
GFR	GFR (ml/min [/1.73 m2 for human])	yes	18	Lognormal	(Fravel et al., 2023)
Papp	Caco-2 apparent permeability coefficient	no	30	Lognormal	estimated
● Fractional blood flows (% or cardiac output)					
QL	Liver debit	yes	32	Normal	(Clewell et al., 1999)
QSL	Slowly perfused tissue debit	yes	30	Normal	(Clewell & Clewell, 2008)
QF	Adipose debit	yes	30	Normal	(Clewell & Clewell, 2008)
QBR	Brain debit	yes	30	Normal	(Clewell & Clewell, 2008)
QTS	Testes debit	yes	30	Normal	(Clewell & Clewell, 2008)
QTH	Thyroid debit	yes	30	Normal	(Clewell & Clewell, 2008)
QBS	Breasts debit	yes	30	Normal	(Clewell & Clewell, 2008)
QR	Rapidly perfused tissues debit	yes	30	Normal	(Clewell & Clewell, 2008)
QSK	Skin debit	no	30	Normal	(Clewell & Clewell, 2008)
QGT	Gut tissue debit	no	30	Normal	(Clewell & Clewell, 2008)
QK	Kidneys debit	no	30	Normal	(Clewell & Clewell, 2008)
● Partition coefficients (tissue-to-plasma)					
PLg	Partition of glucuronide to liver	yes	30	Lognormal	(Clewell et al., 1999)
PSL	Partition of parent BP to slowly perfused tissue	yes	30	Lognormal	(Clewell et al., 1999)
PR	Partition of parent BP to rapidly	yes	30	Lognormal	(Clewell et al., 1999)

	perfused tissue				
PTH	Partition of parent BP to thyroid	yes	20	Lognormal	(Clewell & Clewell, 2008)
PTS	Partition of parent BP to testes	yes	20	Lognormal	(Clewell & Clewell, 2008)
PF	Partition of parent BP to adipose	yes	30	Lognormal	(Clewell et al., 1999)
PBR	Partition of parent BP to brain	yes	30	Lognormal	(Clewell et al., 1999)
PBS	Partition of parent BP to breasts	yes	20	Lognormal	(Clewell & Clewell, 2008)
PL	Partition of parent BP to liver	yes	30	Lognormal	(Clewell et al., 1999)
PSK	Partition of parent BP to skin	no	30	Lognormal	(Clewell et al., 1999)
PGT	Partition of parent BP to gut tissue	no	30	Lognormal	(Clewell et al., 1999)
• Unbound fractions					
FUPBP	Fraction unbound of parent BP in plasma	no	30	Lognormal	estimated
FUPBPG	Fraction unbound of glucuronide in plasma	no	30	Lognormal	estimated
• Glucuronidation kinetics					
Vmax	Maximum velocity (nmol/min/mg)	yes	50	Lognormal	(Thomas et al., 1996)
Km	Michaelis constant (nM)	yes	20	Lognormal	(Thomas et al., 1996)
SFg	Scaling factor for age	yes	69	Lognormal	(Bhatt et al., 2019)

Figure S2. Estimated Papp across the Caco-2 monolayer determined by various techniques.

Squares are results from in vitro experiments with BPF, BPA and BPS, (Kamiya et al., 2020) while crosses refer to predictions for all seven bisphenols by two different QSARs (K: Kamiya et al. (2020) and L: Lanevskij and Didziapetris (2019)). Bisphenols are ordered by ascending MW.

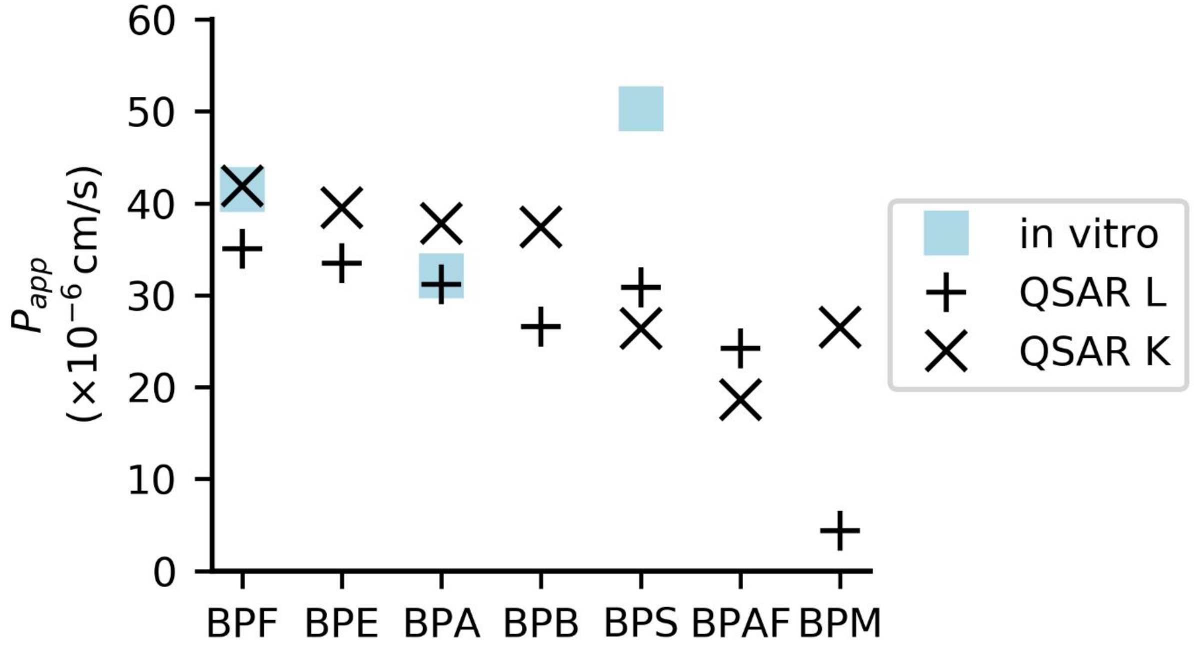

Figure S3. Predicted rates of glucuronidation of bisphenols in human adult liver at physiologically relevant concentrations.

Kinetics of glucuronidation catalyzed by S9 fractions (BPA, BPB, BPE, and BPM, continuous lines) or microsome fractions (BPS, BPF and BPAF, Karrer et al. (2018), dashed lines). Michaelis-Menten kinetics were used to describe the glucuronidation of BPA, BPF and BPS, while substrate-inhibition kinetics were used for BPB, BPE, BPM and BPAF. Bisphenols are ordered by ascending MW.

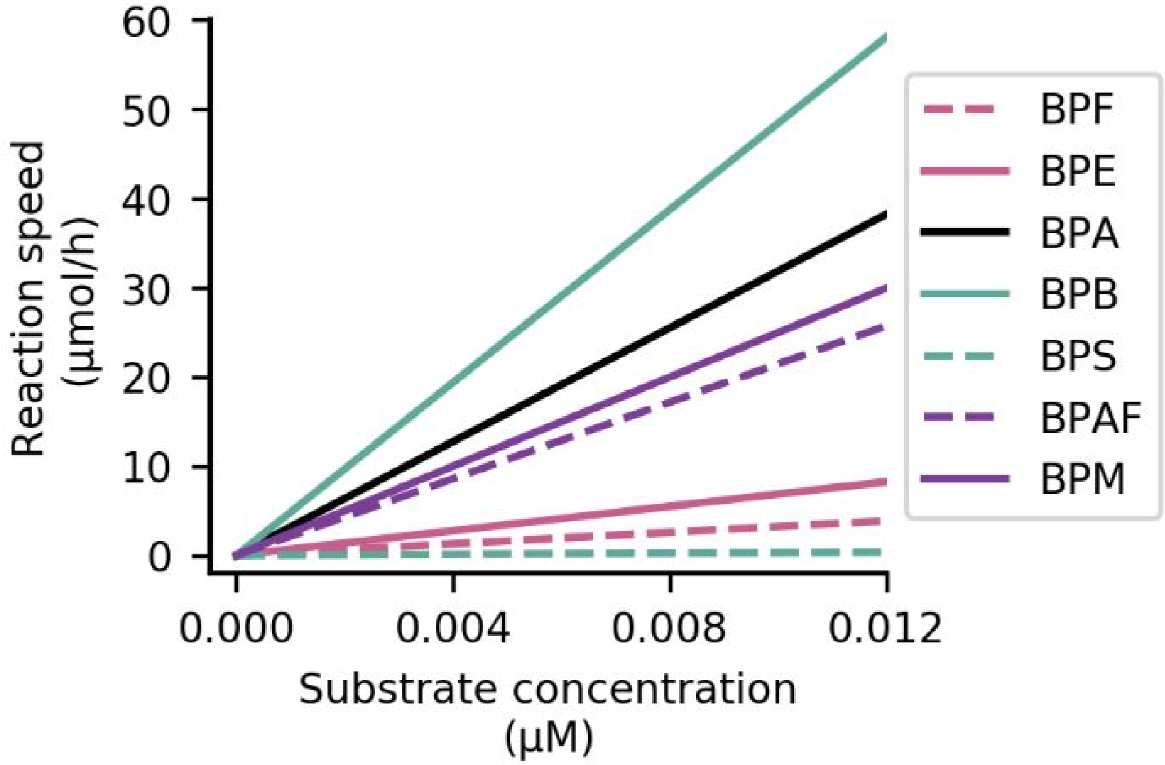

Figure S4: Hepatic glucuronidation kinetics.

BPAF and BPF were measured in rat liver S9 fractions, BPM, BPE, BPA and BPB were measured in human liver S9 fractions.

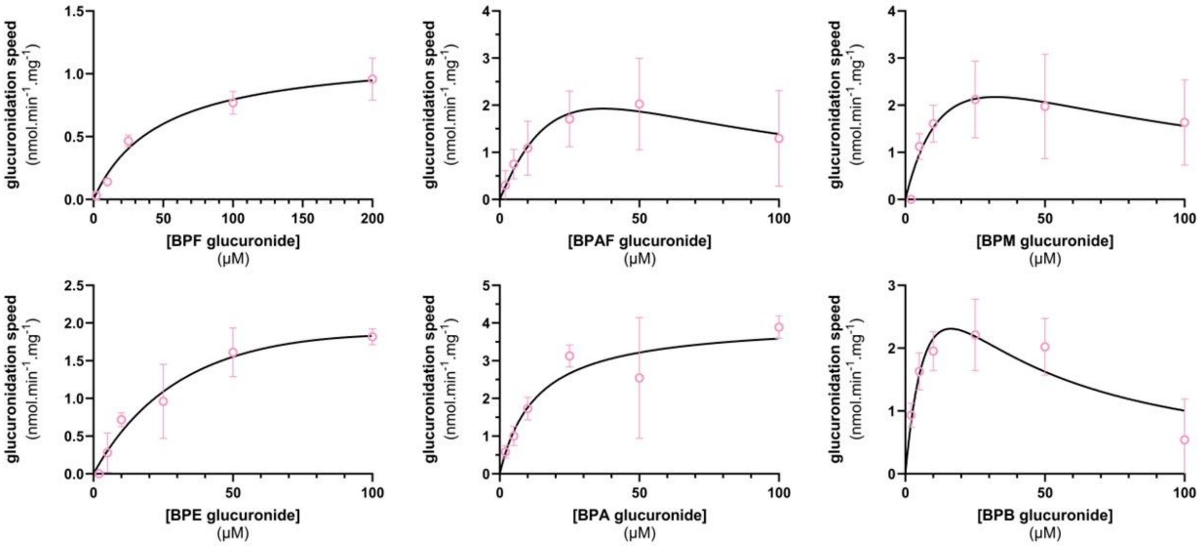

Table S9: Kinetics parameters for hepatic glucuronidation (fitting from GraphPad Prism).

BPF and BPF values were measured in rat liver S9 fractions and used in rat models. BPA, BPB, BPE and BPM were measured in human liver S9 fractions and used in human models. MM: Michaelis Menten, SI: substrate inhibition.

	**BPA**	**BPB**	**BPE**	**BPM**	**BPF**	**BPAF**
Fitted curve	MM	SI	SI	SI	MM	SI
R2	0.90	0.81	0.98	0.92	0.99	0.97
Vmax ($nmol.min^{-1}.mg^{-1}$)	4.06	5.81	3.42	4.89	1.17	7.10
Km (µM)	13.10	12.34	51.31	20.15	47.54	49.68
Ksi (µM)	-	21.48	280.6	51.75	-	27.63

Table S10: EHC rate values.

Bisphenols are ordered by ascending MW.

Sex	BPF	BPE	BPA	BPB	BPS	BPAF	BPM
Male	0.63	0.65	0.67	0.69	0.70	0.81	0.81
Female	0.63	0.66	0.68	0.71	0.73	0.89	0.89

Figure S5: Frequency of sensitivity of parameters across sensitivity analysis.

Analysis was performed in all human models (4) and bisphenols (7) in 4 different compartments (3 organs of interest per physiological model), for exposure scenarios 5 to 8 (Table 1). Dashed horizontal lines indicate the maximum frequency to which the parameters can be found sensitive among the analysis.

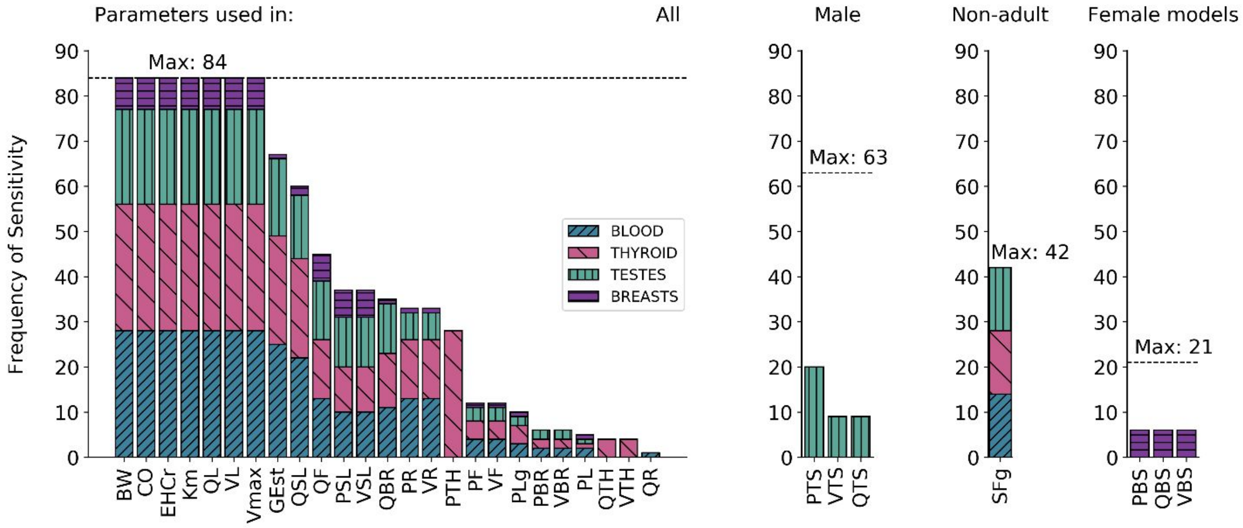

Figure S6: Morris screening exercise for AUC of blood concentration of BPA between t=0 h and t=4 h, with scenarios of exposure 5-8.

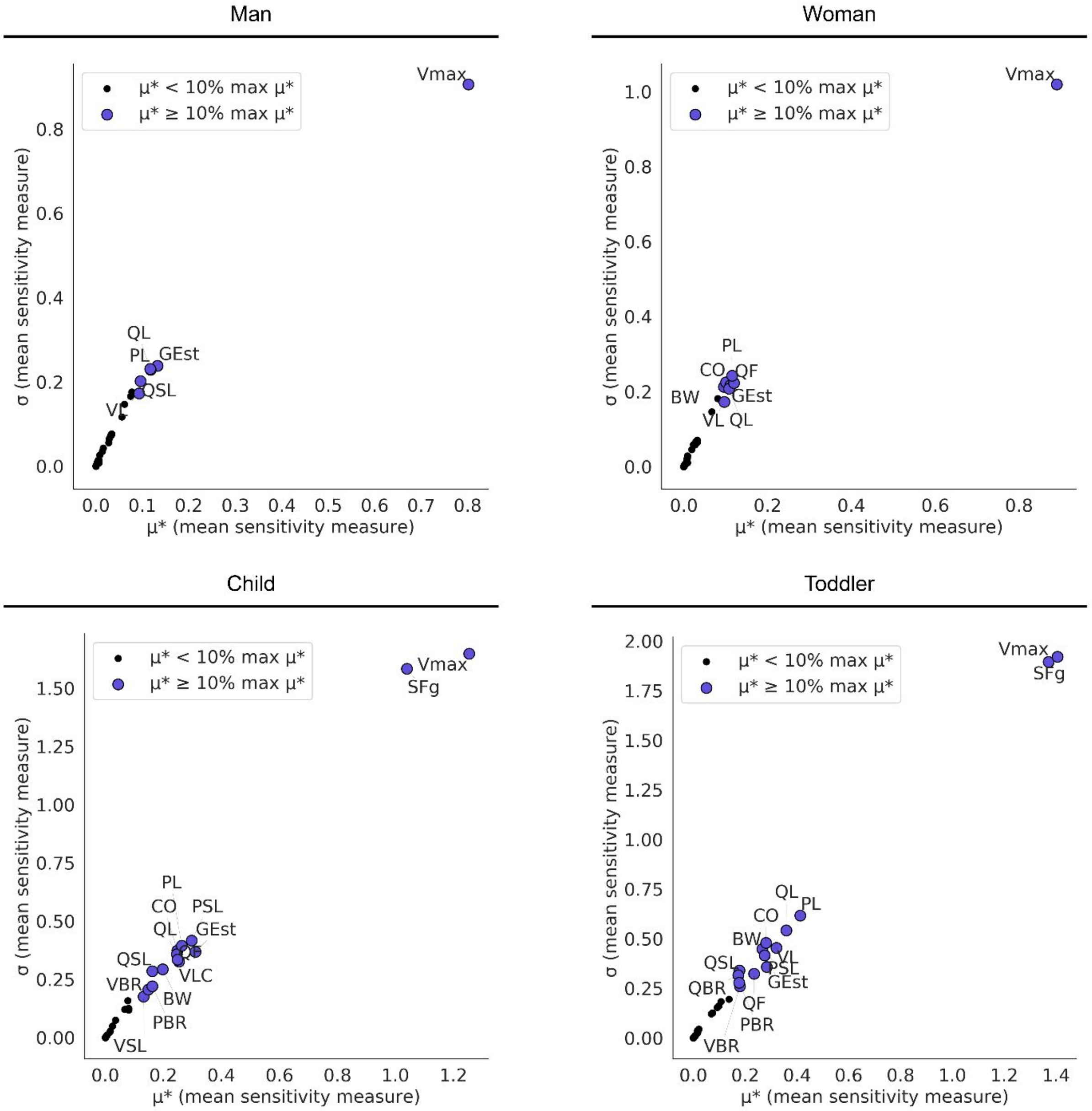

Figure S7: Lowry plots of the eFAST quantitative measures, for AUC of BPA blood concentration between t=0 h and t=4 h, with scenarios of exposure 5-8

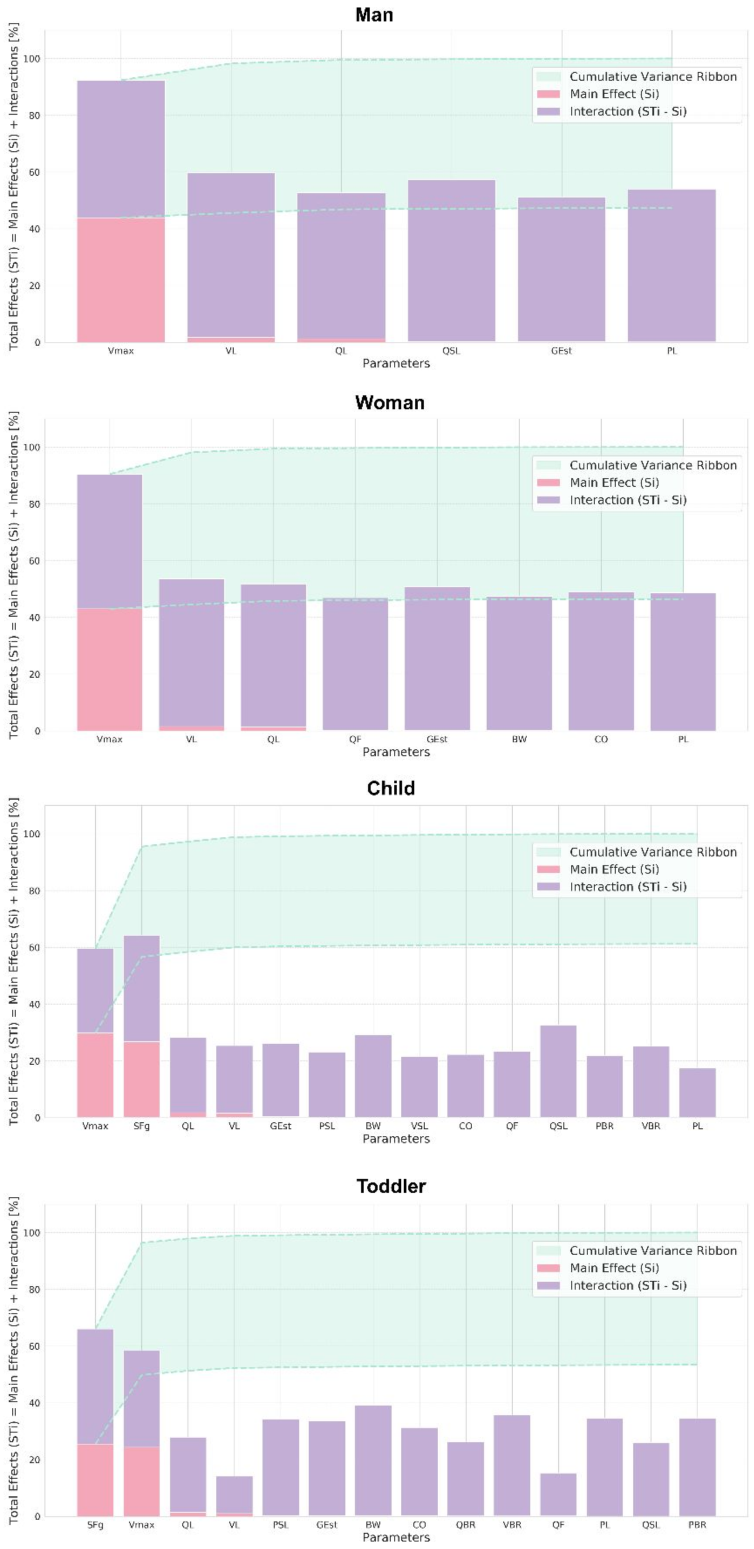

Table S11: Mass balance analysis of excretion

		after 12 h			after 24 h			after 48 h		
		Dose excreted (%)	Dose excreted in urine (%)	Fraction of parent in urine (%)	Dose excreted (%)	Dose excreted in urine (%)	Fraction of parent in urine (%)	Dose excreted (%)	Dose excreted in urine (%)	Fraction of parent in urine (%)
**BPA**	man	96.63	96.62	0.008	99.11	99.1	0.011	99.42	99.41	0.015
	woman	96.27	96.26	0.009	98.85	98.84	0.012	99.2	99.19	0.016
	child	95.88	95.87	0.023	98.36	98.35	0.03	99.12	99.11	0.037
	toddler	96.54	96.53	0.022	98.69	98.68	0.03	99.15	99.14	0.037
**BPAF**	man	85.91	85.89	0.006	95.9	95.88	0.008	97.6	97.58	0.012
	woman	68.67	68.65	0.011	87.39	87.36	0.015	94.34	94.31	0.021
	child	83.57	83.56	0.016	92.56	92.55	0.023	95.19	95.18	0.032
	toddler	85.42	85.41	0.015	94.27	94.25	0.022	96.31	96.29	0.031
**BPB**	man	96.26	96.25	0.003	99.21	99.2	0.004	99.47	99.46	0.005
	woman	95.51	95.5	0.003	98.96	98.95	0.005	99.26	99.25	0.006
	child	95.76	95.75	0.008	98.45	98.44	0.011	99	98.99	0.014
	toddler	96.36	96.35	0.008	98.82	98.81	0.011	99.18	99.17	0.014
**BPE**	man	94.24	94.23	0.063	97.1	97.09	0.086	98.33	98.32	0.108
	woman	93.23	93.22	0.07	96.13	96.12	0.093	97.61	97.6	0.12
	child	90.9	90.89	0.176	95.24	95.23	0.225	98.18	98.17	0.266
	toddler	92.67	92.66	0.171	95.8	95.79	0.22	97.69	97.68	0.266
**BPF**	man	92.22	92.21	0.236	96.02	96.01	0.308	98.38	98.37	0.372
	woman	90.86	90.85	0.252	94.74	94.73	0.333	97.72	97.71	0.414
	child	88	88	0.629	94.93	94.92	0.767	98.98	98.97	0.847
	toddler	89.86	89.85	0.613	94.57	94.56	0.763	98.04	98.03	0.888
**BPM**	man	67.43	67.41	0	88.77	88.74	0	96.39	96.36	0
	woman	49.34	49.32	0.001	73.7	73.67	0.001	89.87	89.84	0.001
	child	69.85	69.84	0.001	87	86.99	0.001	92.22	92.2	0.001
	toddler	67.49	67.48	0.001	87.15	87.13	0.001	94.28	94.26	0.001
**BPS**	man	82.45	82.44	8.005	96.59	96.58	8.419	99.87	99.86	8.499
	woman	78.14	78.13	9.881	94.77	94.76	10.409	99.7	99.69	10.531
	child	82.99	82.98	16.066	97.04	97.03	16.343	99.91	99.9	16.39
	toddler	83.77	83.76	17.001	97.09	97.08	17.504	99.91	99.9	17.593

Figure S8: Predicted concentration profiles in woman (blood, thyroid) and toddler (blood, thyroid, testes) models after single or repeated dose.

Median of 10’000 MC simulations of BPA, BPAF, BPB, BPE, BPF, BPM and BPS in exposure scenarios 6, 8, 10 and 12. Bisphenols are ordered by ascending MW.

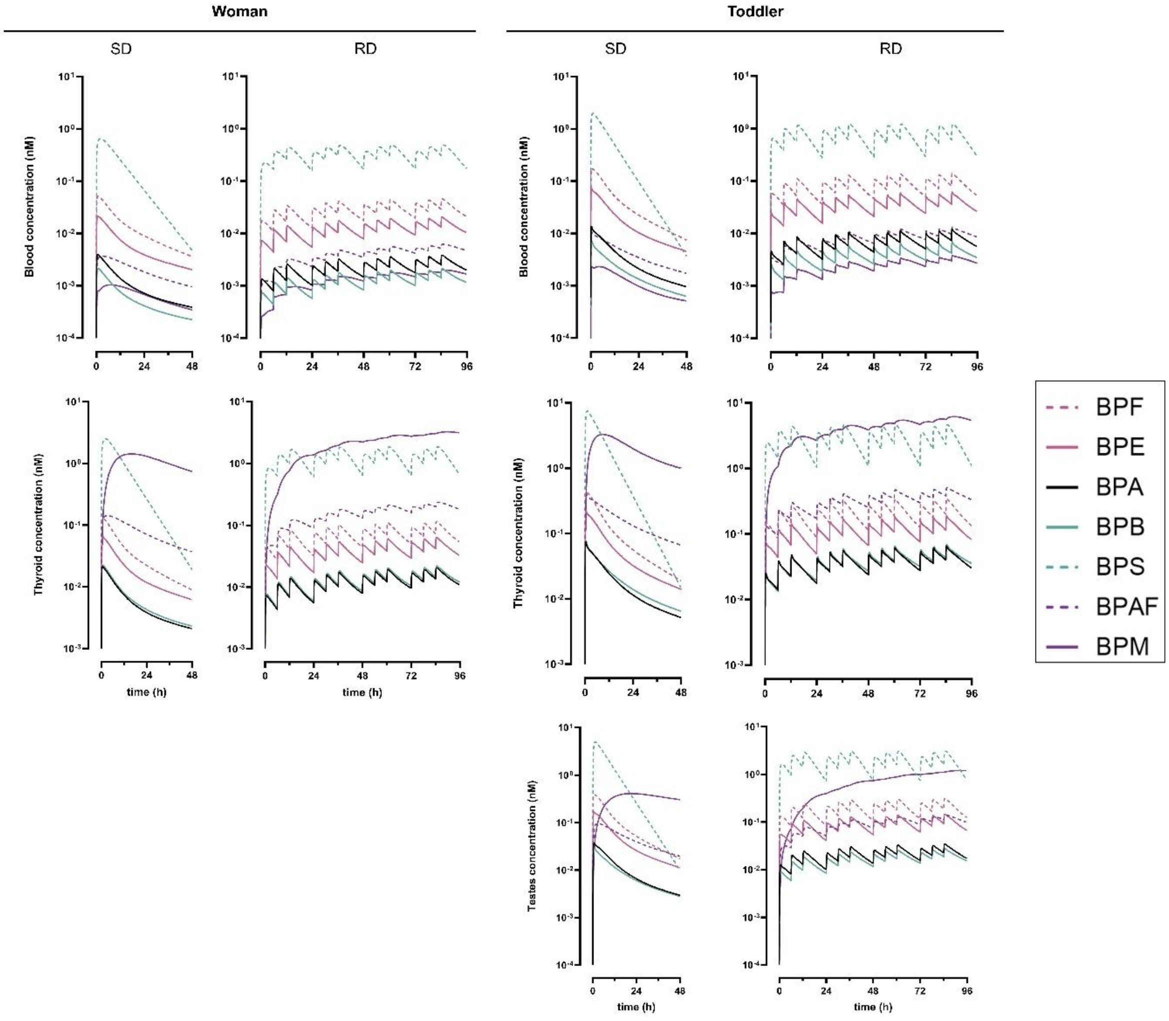

Supplementary information on biomonitoring comparison

Model outputs were generated using Monte Carlo simulations (N = 10’000), incorporating interindividual variability in physiological parameters. Concentrations in various compartments were extracted under the assumption of steady-state conditions, defined here as the 72–96 h interval during repeated dosing simulations. Three summary statistics were computed from the distribution of model outputs: the 5th percentile (lower bound), the median (central tendency), and the 95th percentile (upper bound). For all compartments except urine, the median value across the 72–96 h interval was used to represent each of these percentiles. In the case of urinary excretion, model outputs consisted of the total amount excreted over a 24 h period (i.e., between 72 h and 96 h), which was then divided by the daily urinary volume, adjusted for sex and age based on ICRP (2002) reference values, to compute a predicted concentration.

Biomonitoring studies reporting concentrations of bisphenol analogs in blood, urine, breast tissue, brain, liver, and adipose tissue were identified(Artacho-Cordón et al., 2017; Fernandez et al., 2007; Geens et al., 2012; Kolatorova Sosvorova et al., 2017; Lucarini et al., 2020; Wang et al., 2015; Zhou et al., 2014). The datasets used represent populations exposed under real-world conditions. As such, the frequency and magnitude of exposure among individuals in these studies were neither controlled nor precisely known but reflect chronic environmental exposure that is both highly variable across individuals and specific to particular populations, geographic regions, and time periods. For the purpose of the present model inputs, daily exposure estimates provided by EFSA (2015) were used to simulate repeated oral intake.

Furthermore, the populations included in the biomonitoring studies were not fully adjusted to the physiological models used in the simulations. Although efforts were made to align the data by sex and broad age category (e.g., adult, child), no individual-level adjustment was performed for other physiological or demographic characteristics such as age, body mass index, height, or ethnicity.

Accordingly, these comparisons should not be interpreted as a formal validation of the model’s predictive accuracy, but rather as an exploratory consistency check between model outputs and empirically observed internal concentrations.

Table S12: Biomonitoring comparison

- BPA in blood

Present model (ng/mL)				Biomonitoring measure (ng/mL)				Reference
Model	5%	Median	95%	Population	Minimum	Median	Maximum	
Man	0.0002	0.0005	0.0017	Men	<LLOQ	0.09	8.17	(Kolatorova Sosvorova et al., 2017)

- BPF in blood

Present model (ng/mL)				Biomonitoring measure (ng/mL)				Reference
Model	5%	Median	95%	Population	Minimum	Median	Maximum	
Man	0.0015	0.0054	0.0191	Men	<LLOQ	0.28	6.23	(Kolatorova Sosvorova et al., 2017)

- BPA in breasts

Present model (ng/mL)				Biomonitoring measure (ng/g wet wt)				Reference
Model	5%	Median	95%	Population	Minimum	Median	Maximum	
Woman	0.0025	0.0092	0.0331	Women	< LOD	1.65	3.11	(Keshavarz-Maleki et al., 2021)

- BPA in adipose

Present model (ng/mL)				Biomonitoring measure (ng/g wet wt)				Reference
Model	5%	Median	95%	Population	Minimum	Median	Maximum	
Man	0.0048	0.0212	0.0852	Adults (15 women, 5 men)	<LOQ	3.95 (GM)	~ 20.9	(Wang et al., 2015)
Woman	0.0065	0.0274	0.1083	Men	<LOD	<LOD	3.28	(Artacho-Cordón et al., 2017)
				Women	<LOD	<LOD	0.84	(Artacho-Cordón et al., 2017)
				Women	2.07 (5%)	4.79	11.88 (95%)	(Fernandez et al., 2007)
				9-62 years (3 women, 8 men)	1.12	2.09	12.28	(Geens et al., 2012)

- BPA in brain

Present model (ng/mL)				Biomonitoring measure (ng/g wet wt)				Reference
Model	5%	Median	95%	Population	Minimum	Median	Maximum	
Man	0.0029	0.0107	0.0392	9-62 years (3 women, 8 men)	<LOQ	0.57	2.36	(Geens et al., 2012)
Woman	0.0035	0.0130	0.0498					

- BPAg in liver

Present model (ng/mL)				Biomonitoring measure (ng/g wet wt)				Reference
Model	5%	Median	95%	Population	Minimum	Median	Maximum	
Man	0.0067	0.0170	0.0505	9-62 years (3 women, 8 men)	0.90	1.48	2.77	(Geens et al., 2012)
Woman	0.0070	0.0180	0.0528					

- BPA in liver

Present model (ng/mL)				Biomonitoring measure (ng/g wet wt)				Reference
Model	5%	Median	95%	Population	Minimum	Median	Maximum	
Man	0.0017	0.0056	0.0194	9-62 years (3 women, 8 men)	0.77	1.30	3.35	(Geens et al., 2012)
Woman	0.0022	0.0073	0.0255					

- BPA (total) in urine

Present model (ng/mL)				Biomonitoring measure (ng/mL)				Reference
Model	5%	Median	95%	Population	Minimum	Median	Maximum	
Man	19.9	27.0	36.6	Adults	<LOD	0.72	37.7	(Zhou et al., 2014)
				Men	< LOD	1.76	2.29	(Artacho-Cordón et al., 2017)
Woman	25.5	34.3	46.4	Women	0.51	0.76	1.96	(Artacho-Cordón et al., 2017)
				Women	<LOD	0.74	1.82	(Keshavarz-Maleki et al., 2021)
Child	40.9	54.7	74.5	Children (both sexes)	ND	0.369	3.0358	(Liu et al., 2019)
Toddler	28.6	38.3	51.7	Toddlers (both sexes)	1.22	2.4 (mean)	3.30	(Lucarini et al., 2020)

- BPF (total) in urine

Present model (ng/mL)				Biomonitoring measure (ng/mL)				Reference
Model	5%	Median	95%	Population	Minimum	Median	Maximum	
Man	21.2	28.5	38.6	Adults	<LOD	0.08	212	(Zhou et al., 2014)
Woman	26.8	36.1	48.9					
Child	43.0	57.8	78.4	Children (both sexes)	ND	ND	0.0778	(Liu et al., 2019)
Toddler	29.8	40.2	54.9	Toddlers (both sexes)	1.8	2.7 (mean)	3.61	(Lucarini et al., 2020)

- BPS (total) in urine

Present model (ng/mL)				Biomonitoring measure (ng/mL)				Reference
Model	5%	Median	95%	Population	Minimum	Median	Maximum	
Man	16.6	24.6	36.4	Adults	<LOD	0.13	12.3	(Zhou et al., 2014)
Woman	20.0	31.0	46.4					
Child	29.0	48.3	75.0	Children (both sexes)	ND	0.0188	0.2375	(Liu et al., 2019)
Toddler	20.0	33.7	52.8	Toddlers (both sexes)	2.6	6.41 (mean)	12.02	(Lucarini et al., 2020)

- BPAF (total) in urine

Present model (ng/mL)				Biomonitoring measure (ng/mL)				Reference
Model	5%	Median	95%	Population	Minimum	Median	Maximum	
Child	33.2	45.5	61.8	Children (both sexes)	ND	ND	0.0883	(Liu et al., 2019)

- BPE (total) in urine

Present model (ng/mL)				Biomonitoring measure (ng/mL)				Reference
Model	5%	Median	95%	Population	Minimum	Mean	Maximum	
Toddler	33.2	45.5	61.8	Toddlers (both sexes)	2.7	5.85	9	(Lucarini et al., 2020)

• BPM (total) in urine								
**Present model (ng/mL)**				**Measures of the biomonitoring study**				**Reference**
Model	5%	Median	95%	Population	Minimum	Mean	Maximum	
Toddler	21.9	30.6	42.0	Toddlers (both sexes)	4.38	20.54	49.36	(Lucarini et al., 2020)

Model code (Berkeley Madonna)

```
; PBK model for bisphenols (here BPF) in rat, modeling exposure
described in (Lee et al., 2022)

; Species: Rat
; Molecule: BPF
; Date: 2024.02.19
; Compiled by Hélène Bigonne

{Top model}
   {Reservoirs}

; ST: stomach compartment
   d/dt (ASTBP) = - ST_outtake
      INIT ASTBP = A0DOSEBP
      LIMIT ASTBP >= 0

; GL: gut lumen compartment
   d/dt (AGLBP) =  ST_outtake - GL_outtake + bile_flow_to_gut -FEC
      INIT AGLBP = 0
      LIMIT AGLBP >= 0

; GT: gut tissue compartment
   d/dt (AGTBP) =  GL_outtake - GT_outtake
      INIT AGTBP = 0
      LIMIT AGTBP >= 0

; L: liver compartment
   d/dt (ALBP) =  GT_outtake - L_BPgluc - venL + artL
      INIT ALBP = 0
      LIMIT ALBP >= 0

; B: blood compartment
   d/dt (ABBP) = + venSK + venF + venBR + venL + venSL + venR - artSK
- artR - UR_parent_gfr - artL - artF - artBR - artSL
      INIT ABBP = 0
      LIMIT ABBP >= 0

; BR: brain compartment
   d/dt (ABRBP) = + artBR - venBR
      INIT ABRBP = 0
      LIMIT ABRBP >= 0

; SL: slowly perfused tissues compartment
```

```
   d/dt (ASLBP) = + artSL - venSL
      INIT ASLBP = 0
      LIMIT ASLBP >=0
 ; R: rapidly perfused tissues compartment
   d/dt (ARBP) = - venR + artR
      INIT ARBP = 0
      LIMIT ARBP >= 0

; F: adipose tissue compartment
   d/dt (AFBP) = + artF - venF
      INIT AFBP = 0
      LIMIT AFBP >= 0

; SK: skin compartment
  d/dt (ASKBP) = - venSK + artSK
      INIT ASKBP = 0
      LIMIT ASKBP >= 0

; FECES: fecal compartment
  d/dt (AFECES) = + FEC
      INIT AFECES = 0
      LIMIT AFECES >= 0

; URINE_parent: urine compartment
   d/dt (AURINE_parent) = + UR_parent_gfr
      INIT AURINE_parent = 0
      LIMIT AURINE_parent >= 0

   {Flows}

; gastro_intestinal tract flows
   ST_outtake = kelST *ASTBP
   GL_outtake = ka * CGLBP
   GT_outtake = QGT *CVGTBP

; blood flows
   venL = QL*CVLBP
   artL = QL*CBBP
   artSL = QSL*CBBP
   artBR = QBR*CBBP
   artR = QR*CBBP
   artF = QF*CBBP
   venBR = QBR*CVBRBP
   venSL = QSL*CVSLBP
   venR = QR*CVRBP
```

```
   venF = QF*CVFBP
   venSK = QSK*CVSKBP
   artSK = QSK*CBBP

; excretion flows
   UR_parent_gfr = CUBBP*urineBP
   FEC = AGLBP*KSItransit

   {Submodel "S1"}
      {Reservoirs}

; LBPgluc: liver compartment for BP glucuronide
d/dt (ALBPgluc) = + L_BPgluc - venL_gluc - biliary_excretion
    INIT ALBPgluc = 0
    LIMIT ALBPgluc >= 0

; BILE: bile compartment
d/dt (ABILEBPgluc) = + biliary_excretion - bile_flow_to_gut
    INIT ABILEBPgluc = 0
    LIMIT ABILEBPgluc >= 0

; BBPgluc: blood compartment for BP glucuronide
d/dt (ABBPgluc) = - UR_gluc_gfr - UR_gluc_sec + venL_gluc
    INIT ABBPgluc = 0
    LIMIT ABBPgluc >= 0

; UrineBPgluc: urine compartment for BP glucuronide
d/dt (AUrineBPgluc) = + UR_gluc_gfr + UR_gluc_sec
    INIT AUrineBPgluc = 0
    LIMIT AUrineBPgluc >= 0

      {Flow}
biliary_excretion = EHCr*CVLBPG*QL    ; from liver to bile
bile_flow_to_gut = ABILEBPgluc        ; bile flow to gut lumen
venL_gluc = (1-EHCr)*CVLBPG*QL
L_BPgluc = VmaxLBPFGluc* CVLBP/(Km + CVLBP)
UR_gluc_gfr = urineBP*CUBBPG
UR_gluc_sec=CBBPG*QK*EHCr*(VK/VL)


{Globals}
;======================================================================
; Physiological parameters (rat)
;======================================================================
BW = 0.25       ; bodyweight (kg) (Brown et al., 1997)
```

```
GutSA= 144      ; gut surface area at age 11 weeks (cm2) (Meshkinpour
et al., 1981)
;----------------------------------------------------------------------
; relative tissue volumes
;---(fraction of BW)
VLc = 0.034     ;  liver (Brown et al., 1997)
VRc = 0.0151    ;  rapidly perfused tissues (heart, kidneys, lungs)
(Brown et al., 1997)
VSLc = 0.477    ;  slowly perfused tissue (bone, muscle) (Brown et
al., 1997)
VFc = 0.07      ;  fat tissue (adipose) (Brown et al., 1997)
VPc = 0.074     ;  blood (Brown et al., 1997)
VSKc = 0.197    ;  skin (Brown et al., 1997)
VBRc = 0.006    ;  brain (Brown et al., 1997)
VKc= 0.0073     ; kidneys (Brown et al., 1997)
VSTc = 0.0052   ; stomach (Oatley & Toates, 1969)
VGTc = 0.0162   ; gut tissue (Oatley & Toates, 1969)
;---(mL)
VGLc = 2.18     ; (mL) Vfluid SI lumen (Tanaka et al., 2020)

;----------------------------------------------------------------------
; calculated tissue volumes (L or Kg)
VL = VLc*BW     ; liver
VP = VPc*BW     ; plasma
VR = VRc*BW     ; rapidly perfused tissue
VSL = VSLc*BW   ; slowly perfused tissue
VF = VFc*BW     ; fat tissue
VSK= VSKc*BW    ; skin
VBR = VBRc*BW   ; brain
VST = VSTc*BW   ; stomach
VGT = VGTc*BW   ; gut tissue
VK = VKc*BW
VGL= VGLc/1000  ; gut lumen

;----------------------------------------------------------------------
; blood flow rates
QC = 0.235*BW^(0.75)*60 ; cardiac output (L/h) (Brown et al., 1997)
;--(fraction of QC)(Brown et al., 1997)
QLc = 0.183     ; liver
QFc = 0.07      ; fat
QRc = 0.213     ; rapidly perfused tissue (heart, kidneys, lungs)
QSLc = 0.4      ; slowly perfused tissues (bone, muscle)
QSKc = 0.058    ; skin
QBRc = 0.02     ; brain
QKc = 0.1411
```

```
;-- (ml/min)
QGTc = 7.5       ; (ml/min) gut (Davies & Morris, 1993)

;----------------------------------------------------------------------
; calculated blood flows (L/h)
QL = QLc*QC           ; liver
QF = QFc*QC           ; fat tissue
QR = QRc*QC           ; rapidly perfused
QSL = QSLc*QC         ; slowly perfused
QSK = QSKc*QC         ; skin
QBR = QBRc*QC         ; brain
QK = QKc*QC           ; kidneys
QGT = QGTc/1000*60    ; gut

;======================================================================
; Physicochemical parameters (BPF)
;======================================================================

; molecular weights (g/mol)
MWBP = 200.24         ; parent compound
MWBPGluc = 376.36     ; glucuronidated metabolite

; pKa
pKaBP = 9.56          ; parent compound
pKaBPGluc = 3.26      ; glucuronidated metabolite

; logP
logPBP = 3.30         ; parent compound
logPBPGluc = 2.19     ; glucuronidated metabolite

; logD (parent compound)
logDapical = 3.46
logDbasal = 3.45

; partition coefficients (parent compound) (Punt et al., 2021; Rodgers
& Rowland, 2006)
PLBP = 3.8       ; liver/blood
PFBP = 16.33     ; fat/blood
PRBP = 4.13      ; rapidly perfused tissue/blood
PSLBP = 2.55     ; slowly perfused tissue/blood
PBRBP = 7.19     ; brain/blood
PSKBP = 11.24    ; skin/blood
PGTBP = 7.53     ; gut/blood
```

```
; partition coefficients (metabolite) (Punt et al., 2021; Rodgers &
Rowland, 2006)
PLBPG = 0.11    ; liver/blood

; unbound fractions (Lobell & Sivarajah, 2003; Punt et al., 2021)
FUPBP = 0.091   ; parent compound
FUPBPG = 0.099  ; metabolite
;======================================================================
; Kinetic parameters
;======================================================================

; metabolic parameters
; scaling factors
L=VLc*1000 ; gram liver /kg BW
VLS9 = 165      ; mg S9/ g liver
; hepatic glucuronidation
Km = 47540 ; nM
Vmax = 1.173 ;nmol/min/mg
VmaxLBPFGluc = Vmax*VLS9*60*L*BW

;----------------------------------------------------------------------
; absorption/transfer rates

; intestinal intake (Ka)
Papp= 10^(3-0.0038*MWBP+0.41*logDapical-0.3*logDbasal)/10^(7)
     ;  (cm/s) (Kamiya et al., 2020)
Peff=10^(0.4926*LOG10(Papp) -0.1454)       ; (cm/s) (Sun et al., 2002)
Ka = Peff*GutSA/1000*3600                  ; (L/h)

; gastric emptying (kelst)
GEstc = 25           ; (min) half time of meal in rat stomach (Purdon
& Bass, 1973)
GEst = GEstc /60     ; (h)
LNhalf= -0.69314718       ; LN(1/2)
kelST =  -LNhalf/ GEst     ; (h-1) constant of gastric excretion
; from Ct=C0*e^(-kel*t)

; intestinal transit (KSItransit)
tpassgut = 18             ; (h) passage time through SI+LI (DeSesso &
Jacobson, 2001)
KSItransit = 1/tpassgut          ; (h-1)

;---------------------------------------------------------------------
; EHC parameters
EHCr = 0             ; fraction of BPFG formed subject to EHC
```

```
;----------------------------------------------------------------------
; urinary excretion
GFR = 1.31                 ;ml/min (Davies & Morris, 1993)
urineBP = GFR*(60/1000)    ;L/h

;======================================================================
; Run settings (Lee et al., 2022)
;======================================================================
; oral dose
DOSEBPc = 200000000        ;ng/kg BW
DOSEBP = DOSEBPc*BW        ;ng
A0DOSEBP = DOSEBP/MWBP     ;nmol

;=====================================================================
; Main model calculations/dynamics: BP
;======================================================================
; stomach compartment
CSTBP = ASTBP/VST
;----------------------------------------------------------------------
; gut lumen compartment
CGLBP = AGLBP/VGL
;----------------------------------------------------------------------
; gut tissue compartment
CGTBP = AGTBP/VGT          ; nmol/L ; BPF concentration in gut
CVGTBP = CGTBP/PGTBP       ; partition with gut tissue
;---------------------------------------------------------------------
; liver compartment
CLBP = ALBP/VL
CVLBP = CLBP/PLBP
;----------------------------------------------------------------------
; fat compartment
CFBP = AFBP/VF
CVFBP = CFBP/PFBP
;---------------------------------------------------------------------
; skin compartment
CSKBP = ASKBP/VSK
CVSKBP = CSKBP/PSKBP
;----------------------------------------------------------------------
; rapidly perfused tissue
CRBP = ARBP/VR
CVRBP = CRBP/PRBP
;---------------------------------------------------------------------
; slowly perfused tissue
CSLBP = ASLBP/VSL
CVSLBP = CSLBP/PSLBP
```

```
;----------------------------------------------------------------------
; brain
CBRBP = ABRBP/VBR
CVBRBP = CBRBP/PBRBP
;----------------------------------------------------------------------
; blood compartment
CBBP=ABBP/VP                ; nmol/L (total)
CUBBP=CBBP*FUPBP            ; nmol/L (unbound)
CB1=(CBBP*MWBP)/1000        ; ng/mL (total)
CB2=CB1*FUPBP               ; ng/mL (unbound)

;======================================================================
; Sub-model calculations/dynamics: BPgluc
;======================================================================
; liver
CLBPG = ALBPgluc/VL              ;nmol/L
CVLBPG = CLBPG/PLBPG
; blood
CBBPG = ABBPgluc/VP              ;nmol/L
CUBBPG = CBBPG*FUPBPG            ;nmol/L (unbound)

;======================================================================

{End Globals}
```

References

Artacho-Cordón, F., Arrebola, J. P., Nielsen, O., Hernández, P., Skakkebaek, N. E., Fernández, M. F., Andersson, A. M., Olea, N., & Frederiksen, H. (2017). Assumed non-persistent environmental chemicals in human adipose tissue; matrix stability and correlation with levels measured in urine and serum. *Environmental Research*, *156*, 120-127. https://doi.org/https://doi.org/10.1016/j.envres.2017.03.030

Bhatt, D. K., Mehrotra, A., Gaedigk, A., Chapa, R., Basit, A., Zhang, H., Choudhari, P., Boberg, M., Pearce, R. E., Gaedigk, R., Broeckel, U., Leeder, J. S., & Prasad, B. (2019). Age- and Genotype-Dependent Variability in the Protein Abundance and Activity of Six Major Uridine Diphosphate-Glucuronosyltransferases in Human Liver. *Clin. Pharmacol. Ther.*, *105*(1), 131-141. https://doi.org/10.1002/cpt.1109

Bommarito, P. A., Stevens, D. R., Welch, B. M., Weller, D., Meeker, J. D., Cantonwine, D. E., McElrath, T. F., & Ferguson, K. K. (2023). Temporal trends and predictors of phthalate, phthalate replacement, and phenol biomarkers in the LIFECODES Fetal Growth Study. *Environ. Int.*, *174*, 107898. https://doi.org/10.1016/j.envint.2023.107898

Brown, R. P., Delp, M. D., Lindstedt, S. L., Rhomberg, L. R., & Beliles, R. P. (1997). Physiological parameter values for physiologically based pharmacokinetic models. *Toxicol Ind Health*, *13*(4), 407-484. https://doi.org/10.1177/074823379701300401

Clewell, H. J., Gearhart, J. M., Gentry, P. R., Covington, T. R., VanLandingham, C. B., Crump, K. S., & Shipp, A. M. (1999). Evaluation of the uncertainty in an oral reference dose for methylmercury due to interindividual variability in pharmacokinetics. *Risk Anal*, *19*(4), 547-558. https://doi.org/10.1023/a:1007017116171

Clewell, R. A., & Clewell, H. J. (2008). Development and specification of physiologically based pharmacokinetic models for use in risk assessment. *Regul. Toxicol. Pharmacol.* , *50*(1), 129-143. https://doi.org/10.1016/j.yrtph.2007.10.012

Davies, B., & Morris, T. (1993). Physiological Parameters in Laboratory Animals and Humans. *Pharm. Res.*, *10*(7), 1093-1095. https://doi.org/10.1023/A:1018943613122

DeSesso, J. M., & Jacobson, C. F. (2001). Anatomical and physiological parameters affecting gastrointestinal absorption in humans and rats. *Food Chem. Toxicol.*, *39*(3), 209-228. https://doi.org/10.1016/S0278-6915(00)00136-8

EFSA. (2015). Scientific opinion on the risks to public health related to the presence of bisphenol A (BPA) in foodstuffs. In (Vol. 13, pp. 3978). EFSA Journal.

Fernandez, M. F., Arrebola, J. P., Taoufiki, J., Navalón, A., Ballesteros, O., Pulgar, R., Vilchez, J. L., & Olea, N. (2007). Bisphenol-A and chlorinated derivatives in adipose tissue of women. *Reproductive Toxicology*, *24*(2), 259-264. https://doi.org/https://doi.org/10.1016/j.reprotox.2007.06.007

Fravel, M. A., Ernst, M. E., Webb, K. L., Wetmore, J. B., Wolfe, R., Woods, R. L., Reid, C. M., Chowdhury, E., Murray, A. M., & Polkinghorne, K. R. (2023). GFR Variability, Survival, and Cardiovascular Events in Older Adults. *Kid Med.*, *5*(2), 100583. https://doi.org/10.1016/j.xkme.2022.100583

Gálvez-Ontiveros, Y., Moscoso-Ruiz, I., Almazán Fernández de Bobadilla, V., Monteagudo, C., Giménez-Martínez, R., Rodrigo, L., Zafra-Gómez, A., & Rivas, A. (2023). Levels of Bisphenol A and its analogs in nails, saliva, and urine of children: a case control study [Original Research]. *Front. nutr.*, *10*. https://doi.org/10.3389/fnut.2023.1226820

Geens, T., Neels, H., & Covaci, A. (2012). Distribution of bisphenol-A, triclosan and n-nonylphenol in human adipose tissue, liver and brain. *Chemosphere*, *87*(7), 796-802. https://doi.org/https://doi.org/10.1016/j.chemosphere.2012.01.002

Guiastrennec, B., Sonne, D. P., Bergstrand, M., Vilsbøll, T., Knop, F. K., & Karlsson, M. O. (2018). Model-Based Prediction of Plasma Concentration and Enterohepatic Circulation of Total Bile Acids in Humans. *CPT Pharmacometrics Syst Pharmacol*, *7*(9), 603-612. https://doi.org/10.1002/psp4.12325

Gys, C., Bastiaensen, M., Bruckers, L., Colles, A., Govarts, E., Martin, L. R., Verheyen, V., Koppen, G., Morrens, B., Den Hond, E., De Decker, A., Schoeters, G., & Covaci, A. (2021). Determinants of exposure levels of bisphenols in flemish adolescents. *Environ. Res.*, *193*, 110567. https://doi.org/10.1016/j.envres.2020.110567

Helander, H. F., & Fändriks, L. (2014). Surface area of the digestive tract – revisited. *Scand. J. Gastroenterol.*, *49*(6), 681-689. https://doi.org/10.3109/00365521.2014.898326

ICRP. (2002). Basic anatomical and physiological data for use in radiological protection: reference values. A report of age- and gender-related differences in the anatomical and physiological characteristics of reference individuals. ICRP Publication 89. *Ann. ICRP.*, *32*(3-4), 1-277. https://doi.org/10.1016/S0146-6453(03)00002-2

Jeseta, M., Kalina, J., Franzova, K., Fialkova, S., Hosek, J., Mekinova, L., Crha, I., Kempisty, B., Ventruba, P., & Navratilova, J. (2024). Cross sectional study on exposure to BPA and its analogues and semen parameters in Czech men. *Environ. Pollut.*, *345*, 123445. https://doi.org/10.1016/j.envpol.2024.123445

Jiang, V. S., Calafat, A. M., Williams, P. L., Chavarro, J. E., Ford, J. B., Souter, I., Hauser, R., & Mínguez-Alarcón, L. (2023). Temporal trends in urinary concentrations of phenols, phthalate metabolites and phthalate replacements between 2000 and 2017 in Boston, MA. *Sci. Total Environ.*, *898*, 165353. https://doi.org/10.1016/j.scitotenv.2023.165353

Kamiya, Y., Takaku, H., Yamada, R., Akase, C., Abe, Y., Sekiguchi, Y., Murayama, N., Shimizu, M., Kitajima, M., Shono, F., Funatsu, K., & Yamazaki, H. (2020). Determination and prediction of permeability across intestinal epithelial cell monolayer of a diverse range of industrial chemicals/drugs for estimation of oral absorption as a putative marker of hepatotoxicity. *Toxicol. Rep.*, *7*, 149-154. https://doi.org/10.1016/j.toxrep.2020.01.004

Karrer, C., Roiss, T., Goetz, N. v., Skledar, D. G., Mašič, L. P., & Hungerbühler, K. (2018). Physiologically Based Pharmacokinetic (PBPK) Modeling of the Bisphenols BPA, BPS, BPF, and BPAF with New Experimental Metabolic Parameters: Comparing the Pharmacokinetic Behavior of BPA with Its Substitutes. *Environ. Health Perspect.*, *126*(7), 077002. https://doi.org/10.1289/EHP2739

Keshavarz-Maleki, R., Kaviani, A., Omranipour, R., Gholami, M., Khoshayand, M. R., Ostad, S. N., & Sabzevari, O. (2021). Bisphenol-A in biological samples of breast cancer mastectomy and mammoplasty patients and correlation with levels measured in urine and tissue. *Scientific Reports*, *11*(1), 18411. https://doi.org/10.1038/s41598-021-97864-6

Kolatorova Sosvorova, L., Chlupacova, T., Vitku, J., Vlk, M., Heracek, J., Starka, L., Saman, D., Simkova, M., & Hampl, R. (2017). Determination of selected bisphenols, parabens and estrogens in human plasma using LC-MS/MS. *Talanta*, *174*, 21-28. https://doi.org/10.1016/j.talanta.2017.05.070

Lanevskij, K., & Didziapetris, R. (2019). Physicochemical QSAR Analysis of Passive Permeability Across Caco-2 Monolayers. *J. Pharm. Sci.*, *108*(1), 78-86. https://doi.org/10.1016/j.xphs.2018.10.006

Lee, S., An, K. S., Kim, H. J., Noh, H. J., Lee, J., Lee, J., Song, K. S., Chae, C., & Ryu, H. Y. (2022). Pharmacokinetics and toxicity evaluation following oral exposure to bisphenol F. *Arch. Toxicol.*, *96*(6), 1711-1728. https://doi.org/10.1007/s00204-022-03246-w

Levey, A. S., Inker, L. A., & Coresh, J. (2014). GFR estimation: from physiology to public health. *Am J Kidney Dis*, *63*(5), 820-834. https://doi.org/10.1053/j.ajkd.2013.12.006

Liu, Y., Yan, Z., Zhang, Q., Song, N., Cheng, J., Torres, O. L., Chen, J., Zhang, S., & Guo, R. (2019). Urinary levels, composition profile and cumulative risk of bisphenols in

preschool-aged children from Nanjing suburb, China. *Ecotoxicology and Environmental Safety*, *172*, 444-450. https://doi.org/https://doi.org/10.1016/j.ecoenv.2019.02.002
Lobell, M., & Sivarajah, V. (2003). In silico prediction of aqueous solubility, human plasma protein binding and volume of distribution of compounds from calculated pKa and AlogP98 values. *Mol Divers*, *7*(1), 69-87. https://doi.org/10.1023/b:modi.0000006562.93049.36
Lucarini, F., Krasniqi, T., Bailat Rosset, G., Roth, N., Hopf, N. B., Broillet, M.-C., & Staedler, D. (2020). Exposure to New Emerging Bisphenols Among Young Children in Switzerland. *Int. J. Environ. Res. Public Health.*, *17*(13). https://doi.org/10.3390/ijerph17134793
Lyu, Z., Harada, K. H., Kim, S., Fujitani, T., Hitomi, T., Pan, R., Park, N., Fujii, Y., Kho, Y., & Choi, K. (2023). Temporal trends in bisphenol exposures and associated health risk among Japanese women living in the Kyoto area from 1993 to 2016. *Chemosphere*, *316*, 137867. https://doi.org/10.1016/j.chemosphere.2023.137867
McNally, K., Cotton, R., & Loizou, G. D. (2011). A Workflow for Global Sensitivity Analysis of PBPK Models. *Front Pharmacol*, *2*, 31. https://doi.org/10.3389/fphar.2011.00031
Meshkinpour, H., Smith, M., & Hollander, D. (1981). Influence of aging on the surface area of the small intestine in the rat. *Exp. Gerontol.*, *16*(5), 399-404. https://doi.org/10.1016/0531-5565(81)90061-9
Oatley, K., & Toates, F. M. (1969). The passage of food through the gut of rats and its uptake of fluid. *Psychon. Sci.*, *16*(5), 225-226. https://doi.org/10.3758/BF03332656
Oberle, R. L., Chen, T.-S., Lloyd, C., Barnett, J. L., Owyang, C., Meyer, J., & Amidon, G. L. (1990). The influence of the interdigestive migrating myoelectric complex on the gastric emptying of liquids. *Gastroenterology*, *99*(5), 1275-1282. https://doi.org/10.1016/0016-5085(90)91150-5
OECD. (2021). Guidance document on the characterisation, validation and reporting of Physiologically Based Kinetic (PBK) models for regulatory purposes (H. a. S. Environment, Environment Directorate, Trans.). In (Vol. No. 331). OECD Series on Testing and Assessment.
Punt, A., Pinckaers, N., Peijnenburg, A., & Louisse, J. (2021). Development of a Web-Based Toolbox to Support Quantitative In-Vitro-to-In-Vivo Extrapolations (QIVIVE) within Nonanimal Testing Strategies. *Toxicol. Appl. Pharmacol.*, *34*(2), 460-472. https://doi.org/10.1021/acs.chemrestox.0c00307
Purdon, R. A., & Bass, P. (1973). Gastric and Intestinal Transit in Rats Measured by a Radioactive Test Meal. *Gastroenterology*, *64*(5), 968-976. https://doi.org/10.1016/S0016-5085(73)80009-5
Rodgers, T., & Rowland, M. (2006). Physiologically based pharmacokinetic modelling 2: Predicting the tissue distribution of acids, very weak bases, neutrals and zwitterions. *J. Pharm. Sci.*, *95*(6), 1238-1257. https://doi.org/10.1002/jps.20502
Snipes, R. L. (1997). Intestinal absorptive surface in mammals of different sizes. *Adv Anat Embryol Cell Biol*, *138*, III-VIII, 1-90. https://doi.org/10.1007/978-3-642-60822-3
Sun, D., Lennernas, H., Welage, L. S., Barnett, J. L., Landowski, C. P., Foster, D., Fleisher, D., Lee, K.-D., & Amidon, G. L. (2002). Comparison of Human Duodenum and Caco-2 Gene Expression Profiles for 12,000 Gene Sequences Tags and Correlation with Permeability of 26 Drugs. *Pharm. Res.*, *19*(10), 1400-1416. https://doi.org/10.1023/A:1020483911355
Tanaka, Y., Higashino, H., Kataoka, M., & Yamashita, S. (2020). In Vivo Fluid Volume in Rat Gastrointestinal Tract: Kinetic Analysis on the Luminal Concentration of Nonabsorbable FITC-Dextran After Oral Administration. *J Pharm Sci*, *109*(6), 1863-1866. https://doi.org/10.1016/j.xphs.2020.03.005
Teeguarden, J. G., Twaddle, N. C., Churchwell, M. I., Yang, X., Fisher, J. W., Seryak, L. M., & Doerge, D. R. (2015). 24-hour human urine and serum profiles of bisphenol A: Evidence

against sublingual absorption following ingestion in soup. *Toxicol. Appl. Pharmacol.*, *288*(2), 131-142. https://doi.org/10.1016/j.taap.2015.01.009
Thomas, R. S., Lytle, W. E., Keefe, T. J., Constan, A. A., & Yang, R. S. H. (1996). Incorporating Monte Carlo Simulation into Physiologically Based Pharmacokinetic Models Using Advanced Continuous Simulation Language (ACSL): A Computational Method. *Fundam. Appl. Toxicol.*, *31*(1), 19-28. https://doi.org/10.1006/faat.1996.0072
Waidyanatha, S., Black, S. R., Aillon, K., Collins, B., Patel, P. R., Riordan, F., Sutherland, V., Robinson, V. G., Fernando, R., & Fennell, T. R. (2019). Toxicokinetics and bioavailability of bisphenol AF following oral administration in rodents: A dose, species, and sex comparison. *Toxicol Appl Pharmacol*, *373*, 39-47. https://doi.org/10.1016/j.taap.2019.04.015
Wang, L., Asimakopoulos, A. G., & Kannan, K. (2015). Accumulation of 19 environmental phenolic and xenobiotic heterocyclic aromatic compounds in human adipose tissue. *Environment International*, *78*, 45-50. https://doi.org/https://doi.org/10.1016/j.envint.2015.02.015
Willmann, S., Höhn, K., Edginton, A., Sevestre, M., Solodenko, J., Weiss, W., Lippert, J., & Schmitt, W. (2007). Development of a Physiology-Based Whole-Body Population Model for Assessing the Influence of Individual Variability on the Pharmacokinetics of Drugs. *J. Pharmacokinet. Pharmacodyn.*, *34*(3), 401-431. https://doi.org/10.1007/s10928-007-9053-5
Xi, J., Su, X., Wang, Z., Ji, H., Chen, Y., Liu, X., Miao, M., Liang, H., & Yuan, W. (2023). The associations between concentrations of gestational bisphenol analogues and thyroid related hormones in cord blood: A prospective cohort study. *Ecotoxicol. Environ. Saf.*, *256*, 114838. https://doi.org/10.1016/j.ecoenv.2023.114838
Zheng, Q., Xiao, J., Zhang, D., Li, X., Xu, J., Ma, J., Xiao, Q., Fu, J., Guo, Z., Zhu, Y., Ji, J., & Lu, S. (2024). Bisphenol analogues in infant foods in south China and implications for infant exposure. *Sci. Total Environ.*, *910*, 168509. https://doi.org/10.1016/j.scitotenv.2023.168509
Zhou, X., Kramer, J. P., Calafat, A. M., & Ye, X. (2014). Automated on-line column-switching high performance liquid chromatography isotope dilution tandem mass spectrometry method for the quantification of bisphenol A, bisphenol F, bisphenol S, and 11 other phenols in urine. *Journal of Chromatography B*, *944*, 152-156. https://doi.org/https://doi.org/10.1016/j.jchromb.2013.11.009
